# Multi-ancestry phenome-wide association of complement component 4 variation with psychiatric and brain phenotypes in youth

**DOI:** 10.1186/s13059-023-02878-0

**Published:** 2023-03-07

**Authors:** Leanna M. Hernandez, Minsoo Kim, Pan Zhang, Richard A. I. Bethlehem, Gil Hoftman, Robert Loughnan, Diana Smith, Susan Y. Bookheimer, Chun Chieh Fan, Carrie E. Bearden, Wesley K. Thompson, Michael J. Gandal

**Affiliations:** 1 Department of Psychiatry, Semel Institute, David Geffen School of Medicine, University of California, Los Angeles, Los Angeles, CA 90095 USA; 2 Department of Human Genetics, David Geffen School of Medicine, University of California, Los Angeles, Los Angeles, CA 90095 USA; 3 Program in Neurobehavioral Genetics, Semel Institute, David Geffen School of Medicine, University of California, Los Angeles, Los Angeles, CA 90095 USA; 4 University of Cambridge, Department of Psychiatry, Cambridge Biomedical Campus, Cambridge, CB2 0SZ UK; 5 Population Neuroscience and Genetics Lab, University of California, San Diego, San Diego, CA 92093 USA; 6 Department of Psychiatry, Perelman School of Medicine at the University of Pennsylvania, Philadelphia, PA USA; 7 Lifespan Brain Institute at Penn Med and the Children’s Hospital of Philadelphia, Philadelphia, PA USA; 8 Department of Genetics, Perelman School of Medicine at the University of Pennsylvania, Philadelphia, PA USA

**Keywords:** Schizophrenia, Psychosis, Neuroimaging, Genetics, Gene expression, Complement, Brain

## Abstract

**Background:**

Increased expression of the complement component 4A (*C4A*) gene is associated with a greater lifetime risk of schizophrenia. In the brain, *C4A* is involved in synaptic pruning; yet, it remains unclear the extent to which upregulation of *C4A* alters brain development or is associated with the risk for psychotic symptoms in childhood. Here, we perform a multi-ancestry phenome-wide association study in 7789 children aged 9–12 years to examine the relationship between genetically regulated expression (GREx) of *C4A*, childhood brain structure, cognition, and psychiatric symptoms.

**Results:**

While *C4A* GREx is not related to childhood psychotic experiences, cognition, or global measures of brain structure, it is associated with a localized reduction in regional surface area (SA) of the entorhinal cortex. Furthermore, we show that reduced entorhinal cortex SA at 9–10 years predicts a greater number and severity of psychosis-like events at 1-year and 2-year follow-up time points. We also demonstrate that the effects of *C4A* on the entorhinal cortex are independent of genome-wide polygenic risk for schizophrenia.

**Conclusions:**

Our results suggest neurodevelopmental effects of *C4A* on childhood medial temporal lobe structure, which may serve as a biomarker for schizophrenia risk prior to symptom onset.

**Supplementary Information:**

The online version contains supplementary material available at 10.1186/s13059-023-02878-0.

## Background

Schizophrenia is a highly heritable neurodevelopmental disorder characterized by the presence of hallucinations, delusions, disorganized behavior, and an array of negative symptoms (e.g., flattened affect, reduced motivation) and cognitive deficits. While clinical diagnosis typically occurs in late adolescence or early adulthood, frank psychosis is often preceded by a prodromal period of weeks to years during which gradual changes in cognition, perception, and motivation occur [1]. An estimated 10% of youth between the ages of 9 and 18 experience subclinical psychosis-like experiences (PLEs), including atypical sensory experiences and delusional beliefs [2], which are associated with significantly greater odds of developing schizophrenia in adulthood [2, 3]. One proposed mechanism through which normative developmental processes may contribute to schizophrenia pathophysiology is through atypical brain development and refinement of synaptic connections across childhood and adolescence [4, 5]. A major goal of modern psychiatric research is to identify the genetic and brain-based antecedents of prodromal psychosis, with the ultimate goal of facilitating early intervention, prevention, and better treatment.

The most statistically significant genetic association with schizophrenia is located within the major histocompatibility complex (MHC) on chromosome 6—a region comprised of genes critical to innate and adaptive immunity [6–10]. Subsequent fine mapping of this locus revealed that a significant proportion of this signal can be attributed to multiallelic copy number variation of the complement component 4A (*C4A*) gene [11]. The complement system plays a crucial role in the peripheral immune response [12], and in the brain, it also plays an active role in pruning and facilitating microglial-mediated engulfment of synapses during development [13–16]. These findings have led to the hypothesis that the overactivation of complement genes in the brain may contribute to altered neural development and synaptic pruning, ultimately conferring risk for the phenotypic expression of schizophrenia symptoms.

The *C4* locus encodes for *C4A* and *C4B* genes, which show common but complex structural variation. Each human haplotype carries a range of *C4A* and/or *C4B* copies, present in either long or short forms (i.e., *C4AL*, *C4AS*, *C4BL*, *C4BS*) depending on the presence of a human endogenous retroviral insertion (HERV) in intron 9 [17]. A higher *C4A* copy number is associated with a greater risk for schizophrenia, proportional to its effects on *C4A* expression [11, 18, 19]. *C4A* copy number also shows sex-dependent effects on schizophrenia risk, with stronger effects in males relative to females [18]. Importantly, while recent neuroimaging work suggests that *C4A* also affects cognition and brain structure in the general adult population [20], no study to date has examined the impact of *C4A* on childhood psychosis-related symptoms or brain development.

**Fig. 1:**
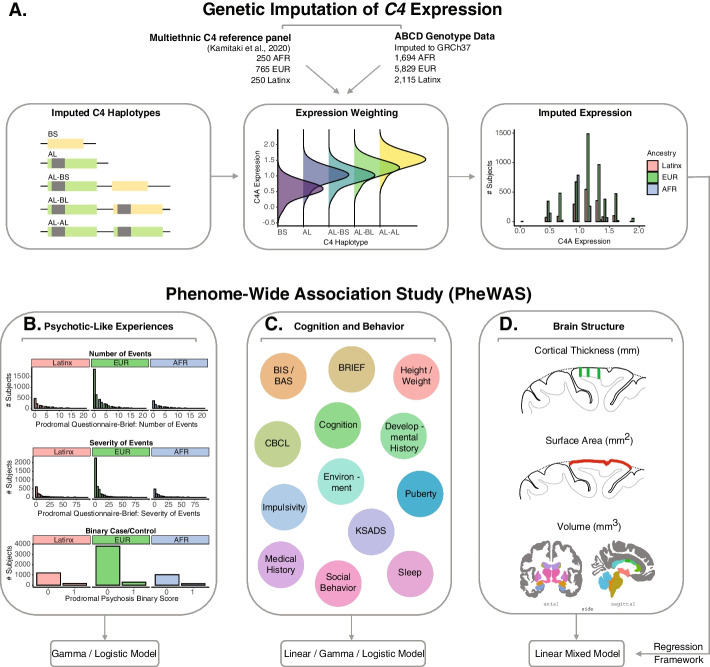
Study design. **A** Individual-level genotype data from ABCD subjects was used to impute *C4* structural alleles using a multi-ancestry reference panel (Kamitaki et al., [18]). *C4A* and *C4B* brain GREx was calculated using previously described weights (Sekar et al., [11]). **B** Generalized linear models were used to test for the associations between *C4A* GREx with psychotic-like experiences (PLE) in ABCD youth. Associations were examined in the whole multi-ancestry cohort, as well as within ancestry- and sex-specific subgroups. **C** We performed a phenome-wide association study (PheWAS) to assess the associations between *C4A* GREx and a host of developmental, cognitive, and behavioral phenotypes (the “Methods” section). **D** Linear mixed-effects models tested the association between predicted *C4A* GREx and neuroimaging measures of cortical thickness, surface area, and volume

Here, we performed a comprehensive, phenome-wide association study between genetically regulated expression (GREx) at the *C4* locus and childhood psychiatric, behavioral, and brain structural phenotypes to elucidate the role of ­*C4* genetic variation on human development (Fig. 1). *C4A* GREx was imputed into a large, ancestrally diverse sample of 9–10-year-old youth who participated in the Adolescent Brain Cognitive Development^SM^ (ABCD) Study [21]. We then assessed whether *C4A* GREx was associated with psychosis-like experiences as well as longitudinal psychiatric and cognitive phenotypes. We find that *C4A* GREx is not broadly related to childhood psychiatric symptoms or cognition in 9–10-year-old youth but is associated with a reduction in entorhinal cortex surface area. Furthermore, we find that entorhinal cortex surface area at 9–10 years of age is predictive of the number and severity of psychotic-like events at 1- and 2-year follow-up visits. Together, this phenome-wide characterization of the developmental effects of *C4A* provides evidence for regional effects on temporal lobe structure, which may be an early biomarker for schizophrenia risk.

## Results

### Genetically regulated expression of *C4A* in the multi-ancestry ABCD youth cohort

Genetic and behavioral data were obtained from a community sample of sociodemographically and ethnically diverse youth who participated in the ABCD Study, an ongoing longitudinal investigation of child development in the USA [22]. Following quality control, including the removal of subjects with low *C4* imputation quality, the sample consisted of 9638 ancestrally diverse youth (1694 African ancestry [AFR]; 5829 European ancestry [EUR]; 2115 Latinx ancestry; Fig. 1A). Imputation accuracy was confirmed by comparing the proportion of youth assigned to each common *C4* structural allele to rates previously reported in a large sample of 765 EUR and 250 AFR ancestry individuals, from which the imputation panel was derived [18] (Additional file 1: Table S1). Subsequent analyses were restricted to 7789 youth with the 5 most common *C4* structural haplotypes (1327 AFR; 4919 EUR; 1543 Latinx; 47.7% female; Additional file 1: Table S2), although in sensitivity analyses, results were concordant across the full spectrum of haplotypes. *C4* structural haplotypes were then used to calculate GREx for *C4A* and *C4B* using previously described weights [11]. We validated the accuracy of *C4* imputation weights for predicting gene expression in ancestrally diverse cohorts using data from 340 AFR, 863 EUR, and 50 Latinx donors from the PsychENCODE Consortium (Additional file 2: Fig. S1). We further validated that these weights were accurate in the subset of 5–15-year-old samples from PsychENCODE, which match the adolescent period investigated in this study. As expected, imputed expression values for *C4A* and *C4B* were negatively correlated (*R* = − 0.25, *P* < 2.2e− 16; Additional file 2: Fig. S2). To control for this, downstream statistical analyses included GREx for both *C4A* and *C4B*.

### Adequacy of statistical power to detect GREx-phenotype associations

Power analyses were conducted in G*Power [23] to determine the range of detectable effects with the current sample size. With an estimated N of 6500, alpha of 0.05, 1 tested predictor, and 11 covariates, we have 95% power to detect effects (Cohen’s *f*^2^) as small as 0.002. As previous research in adults suggests that we can expect to see a significant association between *C4A* GREx and cognitive measures at Cohen’s *f*^2^ = 0.056 [20], power calculations indicate that our study is sufficiently powered to detect small but statistically significant effects, equivalent to a Cohen’s *d* of 0.004.

### No widespread *C4A* associations with psychotic-like experiences or childhood cognition

**Fig. 2:**
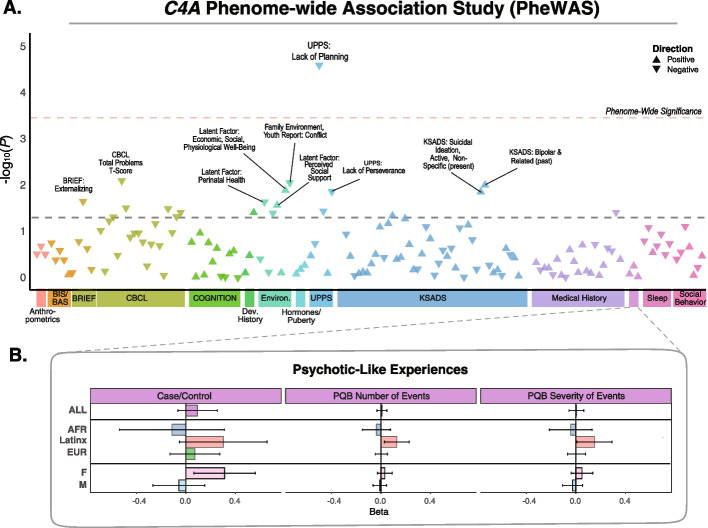
Phenome-wide association between *C4A* GREx and cognitive, behavioral phenotypes in the ABCD cohort. **A** The relationship between *C4A* GREx and 145 phenotypes was tested in the multi-ancestry cohort (*N*’s per phenotype ~ 6000). Increased genetically regulated expression of *C4A* was associated with lower scores on the UPPS-P Impulsive Behavior Scale (Lynam, [27]) lack of premeditation/planning subscale (FDR-corrected *P* = 0.004). Phenotypes are grouped into broad categories by color. Negative or positive associations with *C4A* GREx are indicated by the direction of arrows. The FDR-based threshold for phenome-wide significance is indicated by the red dashed line. **B** Association (absolute 𝛽 ± standard error) between *C4A* GREx and each of the three psychosis variables investigated (binary case/control, PQ-B number PQ-B severity) in the multi-ancestry cohort (i.e., “ALL” youth [as in top panel]), as well as within each ancestry and sex separately. See Additional file 1: Table S3 for the regression model summary statistics. The results by ancestry and sex are shown in Additional file 2: Figs. S3-S8

Models testing associations between *C4A* GREx with rates and severity of PLEs were restricted to unrelated individuals who had complete data for the Prodromal Questionnaire Brief Version [24] (PQ-B) and the Kiddie Schedule for Affective Disorders and Schizophrenia [25] (KSADS-5) (*N* = 6655; 1168 AFR; 4113 EUR; 1374 Latinx). As previous research in clinical high-risk samples indicates that suspiciousness and unusual thought content best predict conversion to psychosis over a 2-year period [26], a binary prodromal psychosis score (PP_bin_) was computed by categorizing youth who endorsed experiencing at least 3 PLEs reflecting suspicious or unusual thought content and significant distress on the PQ-B and whose parents indicated the presence of hallucinations or delusions on the KSADS-5 (*N* = 611 cases, *N* = 6044 controls). We did not observe any significant associations between *C4A* GREx and psychosis symptoms (i.e., PQ-B_sym_, PQ-B_sev_, PP_bin_; Fig. 2, Additional file 1: Table S3, Additional file 2: Figs. S3-S5). As previous work has identified sexually dimorphic effects of *C4A* on the brain and behavior [18, 19], we further examined a potential interaction between sex and *C4A* GREx—which was not significant for any of the psychosis variables tested (Additional file 1: Table S3, Additional file 2: Figs. S6-S8).

In adults and schizophrenia patients, genetic variation within the *C4* locus, as well as in genes that regulate the complement system, has been associated with poorer cognition and memory [20, 28–30]. To assess whether similar effects are present in childhood, we performed a phenome-wide association study (PheWAS) using data from 145 cognitive and psychiatric phenotypes available from the ABCD dataset. In contrast to the findings in adults, *C4A* GREx was not robustly associated with childhood cognition or psychiatric phenotypes (Fig. 2, Additional file 1: Table S3, Additional file 2: Figs. S3-S8). The only association that surpassed correction for multiple testing in the multi-ancestry cohort was between *C4A* GREx and the UPPS-P Impulsive Behavior Scale [27] lack of premeditation/planning subscale (𝛽 = − 0.05, 95% CI [− 0.07, − 0.04], FDR-corrected *P* < 0.004), suggesting that youth with higher *C4A* GREx show less impulsivity and more careful thinking before taking action. This effect was partially mediated by entorhinal cortex size (indirect effect − 0.014, *P* = 0.006).

Additional PheWAS were performed using available data from follow-up time points 1 and 2 years later. There were no FDR-significant associations between *C4A* GREx and PheWAS variables at the 1- or 2-year follow-up (Additional file 1: Tables S4-S5, Additional file 2: Figs. S9-S14). Finally, to assess the convergence between *C4* transcriptomic and genetic associations, regression analyses were performed for the joint model assessing the impact of 14 unique *C4* allelic combinations on childhood PLEs. The *C4* AL:AL haplotype showed nominally significant associations with greater PLEs; however, after Bonferroni correction, the results were no longer significant (Additional file 2: Fig. S15).

### Replicable effects of the *C4* locus on the entorhinal cortex structure

Our PheWAS results above indicate limited detectable effects of *C4* variation on cognitive or psychiatric outcomes in youth. However, in schizophrenia, subtle brain structural changes are known to precede clinical and behavior manifestations [31–33]. To determine whether *C4A* affects childhood brain development, we next tested the association between GREx and measures of cortical thickness (CT), surface area (SA), and volume (VOL) across the whole brain as well as within regional parcellations using the Desikan-Kiliany atlas [34].

**Fig. 3:**
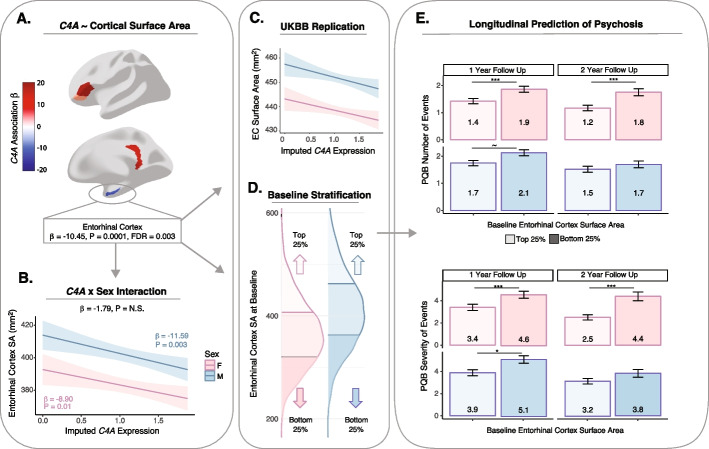
*C4A* GREx is associated with entorhinal cortex surface area and predicts longitudinal psychosis symptoms. **A** Linear regression analyses were performed in the multi-ancestry cohort to test the association between *C4A* GREx and regional brain structure (*N*’s with brain imaging data passing quality control ~ 6500). Brain regions demonstrating a nominally significant association between *C4A* GREx and surface area (mm^2^) are shown in color (*P* < 0.05); only the association with the entorhinal cortex survived FDR correction. The results for all brain regions are provided in Additional File 1: Tables S6-S9. **B** Female and male youths showed a significant effect of *C4A* GREx on reduced entorhinal cortex surface area. **C** Replication of the relationship between *C4A* GREx and entorhinal cortex (EC) surface area in 8357 adult females and 7790 adult males of European ancestry from the UK Biobank. **D** Youths falling in the top and bottom quartiles in terms of baseline entorhinal surface area were identified. **E** Smaller entorhinal cortex surface area (bottom quartile) at baseline predicted greater number and severity of psychosis-like experiences at the 1- and 2-year follow-up time points. The mean number and severity of PLEs are shown inside each bar plot. ~*P* = 0.05, *P < 0.05, **P < 0.01, ***P < 0.001

In the ancestrally diverse ABCD cohort, *C4A* GREx was associated with reduced regional SA of the entorhinal cortex (𝛽 = − 10.45, 95% CI [− 13.13, − 7.76], FDR-corrected *P* = 0.003; Fig. 3A, Additional file 1: Table S6). Furthermore, we found that the relationship between *C4A* GREx and entorhinal cortex was significant for both female and male youths (female: 𝛽 = − 8.90, 95% CI [− 12.46, − 5.34], *P* = 0.01; male: 𝛽 = − 11.6, 95% CI [− 15.56, − 7.63], *P* = 0.003; Fig. 3B). No other brain structure associations were observed after correction for multiple testing (Additional file 1: Tables S7-S9).

To assess the robustness and reproducibility of the impact of *C4A* GREx on entorhinal cortex brain structure, we performed analogous analyses in 16,147 EUR ancestry subjects from the UK Biobank [35]. In the UK Biobank, *C4A* GREx was also significantly associated with entorhinal cortex SA, with the same direction of effect (full sample: 𝛽 = − 5.01, 95% CI [− 7.95, − 2.06], *P* = 0.00087; female 𝛽 = − 5.47, 95% CI [− 8.29, − 0.85], *P* = 0.02; male 𝛽 = − 4.57, 95% CI [− 10.10, − 0.84], *P* = 0.02; Fig. 3C).

The previous analyses show that *C4A* GREx is associated with a localized reduction in entorhinal cortex SA. In psychosis spectrum patients, the size of the entorhinal cortex is a predictor of the severity of delusions and negative symptoms [36, 37]. To assess this, we leveraged longitudinal psychosis data available from the ABCD 1- and 2-year follow-up visits. Due to the differences in overall brain size between male and female youths, analyses were performed in each sex separately and focused on comparing youth in the top and bottom quartile for baseline entorhinal cortex SA (Fig. 3D). We observed a significant difference in both the number and severity of reported PLEs at the 1-year follow-up when comparing between youth in the top and bottom quartiles for entorhinal cortex SA; more specifically, female and male youths with smaller entorhinal cortex SA at baseline reported greater frequency of PLEs and more distressing PLEs 1-year later (Wilcoxon Rank Sum Tests *r* = 0.05–0.09, *P* = 0.0002–0.04; Fig. 3E, Additional file 1: Table S10). At the 2-year follow-up, female youth showed significant differences in PLEs as a function of baseline entorhinal cortex SA; here again, smaller entorhinal cortex SA at baseline was associated with greater frequency and severity of PLEs (*r* = 0.11–0.12, *P* = 0.0002; Fig. 3E, Additional file 1: Table S10). There were no significant associations between entorhinal cortex SA and PLEs in male youth at the 2-year follow-up. Parallel regression analyses modeling entorhinal cortex SA as a continuous predictor were significant in females only, such that smaller entorhinal cortex at baseline predicted greater frequency and severity of PLEs at the 2-year follow-up (PQ-B_sym_ 𝛽 = − 0.001, 95% CI [− 0.002, − 0.0001], *P* = 0.03; PQ-B_sev_ 𝛽 = − 0.002, 95% CI [− 0.003, − 0.0005], *P* = 0.004).

### Effects of *C4A* on the entorhinal cortex are independent of polygenic risk for schizophrenia

Schizophrenia is highly polygenic with thousands of genetic variants of small effect size acting additively to confer risk. To better understand the relationship between genetic risk for schizophrenia at the *C4* locus and additive polygenic risk across the genome (outside the *C4* locus), we computed polygenic risk scores (PRS) in ABCD youth of EUR ancestry (*N* = 3730 unrelated subjects) using the genome-wide association summary statistics of schizophrenia (40,675 cases, 64,643 controls [10]), excluding variants in the MHC region. Similar to previous findings [38], we did not observe a significant association between schizophrenia PRS and psychotic symptoms in youth—nor did we observe an interaction between *C4A* GREx and schizophrenia PRS in predicting PQ-B_sym_, PQ-B_sev_, or PP_bin_ in ABCD youth of EUR ancestry or when examining EUR females and males separately (Additional file 1: Table S11).

**Fig. 4:**
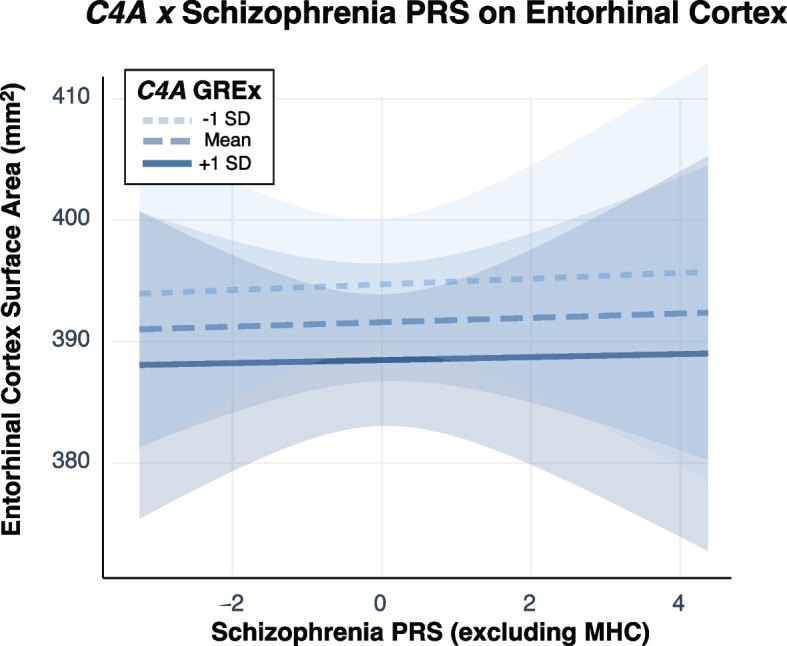
Interactions between *C4A* GREx and polygenic risk for schizophrenia. The interaction between *C4A* GREx and schizophrenia PRS was not a significant predictor of entorhinal cortex surface area in 9–10-year-old youth of European ancestry (*N* = 3349); higher *C4A* GREx was associated with smaller entorhinal cortex surface area across all levels of PRS

Finally, we tested whether schizophrenia PRS—or its interaction with *C4A* GREx—was a significant predictor of brain structure. Surprisingly, schizophrenia PRS showed no association with entorhinal cortex structure, nor was there an interaction with *C4A* GREx, indicating a dissociable effect of complement activation and schizophrenia polygenic burden on this brain structure (Fig. 4, Additional file 1: Tables S11-S14). To confirm this, the association between *C4A* GREx and entorhinal cortex SA remained significant after controlling for schizophrenia PRS (𝛽_*C4A*_ = − 8.88, 95% CI [− 12.46, − .29], *P* = 0.01). We also tested whether the interaction between brain surface area polygenic scores and *C4A* GREx predicted entorhinal cortex SA; the interaction was not significant (*P* > 0.05). Together, these findings suggest that the effects of *C4A* GREx on entorhinal cortex SA are specific to the *C4* locus and are distinct from the effects of genetic liability for schizophrenia and adult brain size.

## Discussion

**Fig. 5:**
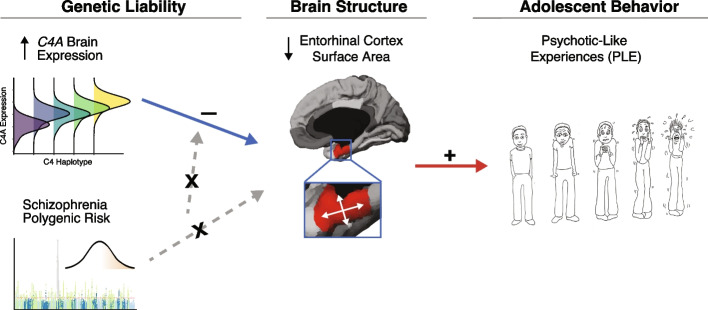
Model for the effects of *C4A* brain expression on childhood brain structure. The effects of increased *C4A* GREx on reduced entorhinal cortex volume in childhood are dissociated from the effects of genome-wide polygenic risk for schizophrenia. Reduced entorhinal cortex surface area in childhood is associated with higher rates of youth-reported psychotic-like experiences (PLEs) in early adolescence, as well as greater severity of events. *Note*: Image under “psychotic-like experiences” taken from the Prodromal Questionnaire Brief Version (PQ-B) to assess the severity of distress associated with psychotic-like events

Here, we conduct a comprehensive multi-ancestry, phenome-wide association study of complement component 4 (*C4*) variation with childhood cognitive, psychiatric, and neuroimaging phenotypes. We find that genetically regulated expression of *C4A* is not associated with broad psychiatric symptoms or brain structure in 9–10-year-old children, but rather shows localized and replicable effects on the reduced regional surface area of the entorhinal cortex. Importantly, this *C4A*-mediated reduction in the entorhinal cortex surface area at 9–10 years of age predicted higher rates and greater severity of psychosis-like experiences at follow-up time points 1 and 2 years later, indicating that the effects of *C4A* on temporal lobe structure may be a biomarker for schizophrenia risk prior to symptom onset (Fig. 5).

Despite having sufficient power to detect associations with small effect sizes, we find that predicted *C4A* expression is not robustly associated with psychiatric symptoms or cognition in 9–10-year-old youth. These null results are surprising given the strength of the association between *C4A* and schizophrenia risk [11]. However, these negative findings comport with recent studies assessing the extent to which polygenic risk for schizophrenia is associated with broad psychiatric phenotypes in the ABCD cohort, which have found limited associations with fluid cognition and an index of binary psychosis severity in ABCD youth of European ancestry [39, 40]. Nevertheless, we identified a significant inverse association between predicted *C4A* expression and lack of planning on the UPPS-P Impulsive Behavior Scale [27]. Higher scores on this subscale are indicative of a tendency not to plan ahead; thus, we find that *C4A* brain expression is related to less severe symptomatology. The effects of schizophrenia genetic risk at the *C4* locus and across the genome may exert more extensive effects on cognition and behavior during later developmental periods (e.g., adolescence and early adulthood), when clinically significant features of psychosis and schizophrenia are more likely to occur. We speculate that the developmental timing and tempo of synaptic pruning may be an important contributor to the emergence of psychiatric symptoms, such that increased pruning during early childhood may be developmentally advantageous until reaching a threshold of “over” pruning later in life.

In addition to the robust genetic association signal observed between schizophrenia and the major histocompatibility complex (MHC) locus, this region also shows associations with depression [41, 42] and to a weaker extent bipolar disorder [7, 43]. However, in both depression and bipolar disorder, dissecting the MHC signal using fine mapping techniques suggests that the observed associations are not driven by genetic variation in C4 haplotypes and arise from other variants in the region [44, 45]. Our null phenome-wide association study (PheWAS) testing the effects of the *C4* locus on childhood psychiatric phenotypes agree these findings, suggesting that other sources of genetic variation apart from the C4 locus are likely influencing childhood cognition and behavior. Further large-scale studies will be needed to disentangle the genetic signal between MHC locus and diverse psychiatric disorders, and to examine the effects of other MHC-associated variants on neurodevelopmental trajectories.

In this demographically diverse multi-ancestry cohort, we observed a regionally localized association between *C4A* GREx and surface area of the entorhinal cortex. Our entorhinal cortex findings are consistent with the extant literature implicating medial temporal lobe structures in the pathophysiology of schizophrenia [46–48]. Indeed, in vivo neuroimaging has consistently found atypical volume of medial temporal lobe structures in patients with psychotic disorders relative to controls [49–51], and several postmortem studies have found aberrant entorhinal cortex cytoarchitecture in schizophrenia patients, suggestive of either atypical neuronal migration or altered pruning during neurodevelopment [52–56].

The entorhinal cortex serves as a relay for corticohippocampal interactions, serving as the major input and output structure of the hippocampus [57]. Recent high-field magnetic resonance imaging (MRI) research in humans suggests an anteroposterior division of entorhinal cortex functional connectivity, with anterior regions connected to anterior temporal brain areas critical for recognition memory, emotional processing, and social cognition and posterior regions connected to posterior medial brain areas that support episodic memory and spatial navigation [58, 59]. Because the entorhinal cortex receives afferent projections from multiple cortical and subcortical brain regions and is the primary source of information to the hippocampus [60], disruptions to this region during development have the potential to affect both the integration of sensory information and memory [58, 61, 62]. In line with this hypothesis, previous work has shown that smaller entorhinal cortex volume is a predictor of cognition and negative/disorganized symptoms in psychosis spectrum youth [37]. Similarly, we find that reduced entorhinal cortex surface area at 9–10 years of age predicts greater number and severity of psychosis-like events 1 and 2 years later. As brain volume is a function of surface area and cortical thickness, our findings of smaller surface area in youth with high genetic risk for schizophrenia at the *C4* locus help to more precisely identify potential morphometric biomarkers predictive of psychosis risk.

Our *surface area*-specific findings in preadolescent youth contrast with adult neuroimaging studies, which have typically found more widespread reductions in *cortical thickness* in schizophrenia patients [63] and in clinically high-risk individuals who transition to psychosis [31]--although we did replicate the effects of *C4A* GREx on entorhinal cortex surface area in adults subjects from the UKBiobank. These contradictory findings may be explained by developmental differences in structural neuroimaging parameters. Recent neuroimaging studies have shown that cortical surface area continues to expand throughout the postnatal period and early childhood [64], while adolescence and early adulthood are characterized by nonlinear decreases in cortical thickness [65], likely driven by developmentally regulated alterations in cortical myelination and pruning. Thus, genetic risk for schizophrenia may have distinct effects on diverse aspects of brain structure at different developmental periods. Importantly, *C4A* cis-eQTLs (i.e., genomic loci that explain variation in gene expression) have been identified in the fetal brain [66], suggesting that the effects of *C4A* GREx on brain morphology may begin early in development. We therefore hypothesize that the observed effects of *C4A* GREx on the cross-sectional entorhinal cortex surface area in children stem from early alterations in brain development and that future studies of the effects of *C4A* on brain development are likely to observe associations with morphological metrics in flux within a particular developmental window (e.g., adolescent cortical thickness).

Notably, the effects of predicted *C4A* GREx on entorhinal cortex surface area are independent and dissociable from genome-wide polygenic risk for schizophrenia. We provide several lines of evidence to support this conclusion. First, polygenic risk scores (PRS) trained using weights from the largest available GWAS of schizophrenia [10] failed to predict entorhinal cortex surface area in youth of European ancestry. Second, when examining the interaction between *C4A* GREx and PRS, we find no evidence that schizophrenia PRS moderates the relationship between *C4A* GREx and entorhinal cortex surface area, and finally, the observed relationship between *C4A* GREx and entorhinal cortex surface area remains significant even when controlling for the effects of PRS. These findings indicate that the observed effects on the entorhinal cortex surface area are exclusive to genetic risk for schizophrenia at the *C4* locus and are distinct from the effects of schizophrenia polygenic risk.

This study has several important limitations. First, our analyses were restricted to youth within the early to mid-adolescent age range. It remains unknown the extent to which *C4A* GREx may affect neurodevelopment during infancy and very early childhood, as well as during adolescence, when many disorders of mental health—including schizophrenia—begin to emerge. Second, our PheWAS analyses were limited to a single neuroimaging modality, and it is unclear whether genetic risk for schizophrenia on the *C4* locus may impact measures of brain functional and structural connectivity, as well as the developmental timing with which these associations may occur. Third, while there are many environmental variables that are likely to influence brain and behavioral development, the current study did not examine the relationship between *C4A* GREx and environmental factors known to increase the risk for schizophrenia. It will be crucial for future studies to examine the impact of *C4A* expression on trajectories of brain development using multimodal neuroimaging techniques and incorporating potential interactions with environmental moderators.

## Conclusions

In sum, the current study reports the first multi-ancestry phenome-wide association of genetic variation with the *C4* locus in children. Our findings indicate that increased *C4A* brain expression in youth is associated with a localized reduction in the size of the entorhinal cortex that is independent of polygenic risk for schizophrenia. Overall, these data help to identify genetically driven brain-based biomarkers predictive of psychosis risk. Ultimately, a comprehensive understanding of the genetic and environmental precursors to psychosis will require longitudinal indices of brain development across the transition to adolescence and young adulthood, when clinically significant behavioral manifestations of psychiatric disorders often begin to emerge.

## Methods

### Participants

Data were obtained from the Adolescent Brain and Cognitive Development (ABCD) Study, an ongoing longitudinal investigation of brain and behavioral development in 11,878 children in the United States of America (USA). Youth were recruited primarily from schools within 21 catchment areas chosen to represent the ethnic and sociodemographic diversity of the larger US population [22]. Analyses were performed using de-identified phenotypic data from the baseline time point obtained from the ABCD 3.0 National Data Archive release (DOI 10.15154/1519007). Analyses were restricted to youth with available genetics data. See Additional file 1: Table S1 for subject characteristics.

### Genotyping and imputation

Genotyping for the ABCD dataset was performed at the Rutgers University Cell and DNA Repository using the Affymetrix NIDA Smokescreen Array (RUCDR). Quality control was performed by the RUCDR and the ABCD Data Analysis and Informatics Core (DAIC) and included filtering for call signals and rates, and processing through the Ricopili pipeline [67]. Genotyping data was merged with data from the 1000 Genomes Project, and ancestry was assigned using the *k*-nearest neighbors classification with the first four genetic principal components.

*C4* structural alleles were imputed for the ABCD dataset using Beagle4.1 [68] with the latest multi-ancestry *C4* imputation reference panel composed of 1265 samples of diverse ancestry [18]. *C4A* and *C4B* genetically regulated expression (GREx) was predicted using previously described expression weights [11]. *C4* structural allele dosages were multiplied by the following coefficients to compute GREx:

$$C4A\ \textrm{GREx}=\left(0.47\times \textrm{AL}\right)+\left(0.47\times \textrm{AS}\right)+\left(0.20\times \textrm{BL}\right)$$

$$C4B\ \textrm{GREx}=\left(1.03\times \textrm{BL}\right)+\left(0.88\times \textrm{BS}\right)$$

Downstream analyses were restricted to samples of African (AFR), European (EUR), and Latinx ancestries as the imputation panel was composed of those individuals, and *C4* imputation accuracy (based on probabilistic dosage) was lower for Southeast Asian and East Asian. Individuals with an average imputed probabilistic dosage < 0.7 were excluded from subsequent analyses. To further ensure the use of high-quality imputed data, analyses were restricted to individuals with the five most common *C4* structural haplotypes (AL, AL-AL, AL-BL, AL-BS, BS) resulting in a final sample of 7789 youth (see Additional file 1: Table S2).

For the UK Biobank, chromosome 6 of the phased haplotype data (i.e., ukb_hap_chr6_v2.bgen) was used to impute *C4* structural alleles. Beagle4.1, with the multi-ancestry *C4* reference panel, was used for imputation, as was done for the ABCD dataset.

### Polygenic risk prediction

Polygenic risk scores (PRS) were calculated for EUR subjects. Genotype imputation was performed on the Michigan Imputation Server [69] using the TOPMed reference panel. Quality control included filtering for genotyping rate > 0.99, sample missingness < 0.01, Hardy-Weinberg Equilibrium *P* > 1 × 10^−6^, minor allele frequency > 1%, and imputation score > 0.8. GCTA v1.93.31 [70] was used to compute the genetic relationship matrix; a relatedness cutoff of 0.05 was applied. Genetic variants in the major histocompatibility complex (MHC) locus were excluded prior to PRS calculation. PRS were generated using summary statistics from the largest available genome-wide association study of schizophrenia [10] (40,675 cases, 64,643 controls) and adult brain surface area [71] (33,992 subjects) in PRS-CS [72], applying the LD reference panel from the 1000 Genomes Project phase 3 samples and the recommended global shrinkage parameter for highly polygenic traits (phi = 1e−2). Analyses assessing the interaction between *C4* expression and PRS were restricted to youth with common *C4* structural haplotypes (AL, AL-AL, AL-BL, AL-BS, BS), resulting in a final sample of 3730 EUR youth (female *N* = 1737; male *N* = 1993) and 7,715,663 genetic variants.

### Neuroimaging data acquisition and preprocessing

Details of the ABCD magnetic resonance imaging (MRI) protocol have been described previously [73]. Briefly, imaging data were collected on either a Siemens Prisma, Phillips, or GE 750 3T scanner using 32-channel head or 64-channel head/neck coils. T1-weighted scan parameters were as follows: Siemens - matrix size 256 × 256, 176 slices, FOV 256 × 256, resolution (mm) 1 × 1 × 1, TR 2500 ms, TE 2.88 ms, flip angle 8°, total scan time 7:12; Phillips - matrix size 256 × 256, 225 slices, FOV 256x240, resolution (mm) 1 × 1 × 1, TR 6.31 ms, TE 2.9 ms, flip angle 8°, total scan time 5:38; and GE - matrix size 256 × 256, 208 slices, FOV 256 × 256, resolution (mm) 1 × 1 × 1, TR 2500 ms, TE 2 ms, flip angle 8°, total scan time 6:09.

Tabulated data representing T1-weighted measurements of subcortical volume (VOL), cortical thickness (CT), and surface area (SA) as derived from the Desikan parcellation atlas [34] in FreeSurfer v5.3 (http://surfer.nmr.mgh.harvard.edu/) were downloaded from the NDA. ABCD-recommended imaging inclusion criteria were followed to ensure the inclusion of high-quality MRI scans, and this excluded participants with serious MR findings and participants whose T1-weighted images or FreeSurfer parcellations failed to pass ABCD DAIC quality control. Regional MRI metrics were averaged across hemispheres.

### Phenotypic data

Psychotic experiences were assessed with the Prodromal Questionnaire Brief Version [24] (PQ-B) and the Kiddie Schedule for Affective Disorders and Schizophrenia [25] (KSADS-5). The PQ-B is a 21-item child-report questionnaire designed to measure the presence and severity of psychotic-like experiences (PLEs) in childhood. PQ-B symptom scores (PQ-B_sym_) were calculated as the total number of endorsed PLEs; severity score (PQ-B_sev_) was calculated as the child-reported level of distress (range 1–5) across all endorsed items. In line with previous research indicating that unusual thought content and suspiciousness are most predictive of psychosis risk [26], a binary score (PP_bin_) was developed by defining prodromal psychosis “cases” as youth who endorsed experiencing at least 3 PLEs reflecting suspicious or unusual thought content (i.e., PQ-B items 1, 4, 5, 7, 8, 11, 12, 13–18), significant distress related to these experiences (i.e., PQ-B distress scores > 6 across items 1, 4, 5, 7, 8, 11, 12, 13–18), and whose parents indicated the child experienced hallucinations, delusions, and associated psychotic symptoms on the KSADS-5 assessment; youth not meeting these criteria served as the “control” group. Cognitive and psychiatric variables from the reported *C4* phenome-wide association study (PheWAS) can be found in Additional file 1: Table S3.

Rates and severity of childhood PLEs were non-normally distributed, with the majority of youth reporting zero or few PLE symptoms (PQ-B_sym_; M = 2.55, SD = 3.52) and endorsing low levels of PLE severity (PQ-B_sev_; M = 6.09, SD = 10.39; Fig. 1B).

### Statistical analyses

Generalized linear models with gamma family and log link were run using the *glm* package in R version 3.6.3 to test the association between *C4* GREx and continuous measures of psychotic experiences (i.e., PQ-B_sym,_ PQ-B_sev_), as these variables demonstrated a right-skewed distribution. The association between *C4* GREx and PP_bin_ was tested via logistic regression with binomial family using the *glm* package in R. Fixed effects included predicted *C4A* and *C4B* GREx, age, socioeconomic status (SES; taken as the average of parent education and income at the baseline time point), four genetic principal components, sex, and site ID. Statistical models run in related individuals using family ID as a random effect failed to converge; thus, analyses were restricted to one randomly selected subject from each family.

Models testing multivariable phenome-wide associations were also restricted to one individual per family. For normally distributed variables, linear models were fitted, while for non-normally distributed variables, generalized linear models with gamma distribution and log link were fitted. *C4A* and *C4B* GREx, age, SES, four genetic principal components, sex, and site ID were specified as fixed effects. Benjamini-Hochberg false discovery rate (FDR) correction was used to account for the number of variables tested at each time point (i.e., baseline, 1-year follow-up, 2-year follow-up). The significance of covariates of interest was assessed using the likelihood ratio test.

To test the association between *C4* GREx and neuroimaging indices of brain volume, cortical thickness, and surface area, linear mixed models were run via the *lme4* package in R. *C4A* and *C4B* GREx, age, four genetic principal components, SES, and sex were entered as fixed effects; MRI device serial number and family ID were entered as random effects. To control for global brain effects, whole brain volume, mean cortical thickness, or mean surface area were also included as covariates for respective models. The significance of covariates of interest was assessed using the likelihood ratio test, and FDR correction was used to account for the number of variables tested within each imaging measure (i.e., VOL, CT, SA).

Sex differences in the effects of *C4* GREx on brain and behavioral phenotypes were tested by including an interaction term between *C4A* and *C4B* GREx and sex in statistical models. Follow-up analyses were performed in each sex separately.

The total number of participants in each analysis (i.e., those with complete PQ-B_sym,_ PQ-B_sev_, PP_bin_, phenotype, and/or brain-imaging data) is provided in Additional file 1.

Plots were generated using R packages *ggpubr*, *ggplot*, and *ggesg*. PheWAS results were plotted in R by modifying publicly available code from Dr. Yoonjung Joo (https://rpubs.com/helloyjjoo/pheWAS_connectome).

### Replication in the UK Biobank

The UK Biobank resource was accessed through application number 38029. Brain imaging data was acquired for 18,361 unrelated individuals with European ancestry. Haplotypes at the *C4* locus were imputed as described for the ABCD dataset. After further restricting to individuals with the five most common *C4* haplotypes (AL, AL-AL, AL-BL, AL-BS, BS), a total of 16,147 individuals remained in the final analyses. A linear model was then fitted between entorhinal cortex surface area and *C4A* GREx, using *C4B* GREx, sex, age at scan, average cortex surface area, location of head in the scanner (data fields 25756 to 25759), first four genotype PCs, and scan sites as covariates. A null model was also fitted with only covariates but not *C4A* GREx. The statistical significance of the association was evaluated by performing a likelihood ratio test. The analysis was performed in females (*N* = 8357) and males (*N* = 7790) separately, without using sex as a covariate.

### Validation of expression weights for C4 genes in PsychENCODE

Previously harmonized genotype array and frontal cortex RNA-seq data from PsychENCODE [19, 74] consisted of uniformly processed data from six studies: BipSeq, LIBD_szControl, CMC_HBCC, CommonMind, BrainGVEX, and UCLA-ASD. We imputed *C4* alleles in six studies from PsychENCODE separately in the same manner as the ABCD dataset using Beagle4.1 with the latest multi-ancestry *C4* imputation reference panel. This analysis consisted of 340 AFR, 863 EUR, and 50 Latinx samples. *C4* expression was then predicted using previously described expression weights [11], which was associated with observed *C4* gene expression (Additional file 2: Fig. S1). Observed *C4* gene expression was strongly associated with corresponding predicted gene expression, except for *C4B* gene expression in AFR samples (*P* > 0.05); *C4A/C4B* expression weights accurately predicted childhood and adolescent *C4A* expression in EUR PsychENCODE samples between the ages of 5-15 (*P* < 0.05).

## Supplementary Information

**Additional file 1: Table S1.** Subject characteristics as assessed at the baseline timepoint in youth with the 5 most common C4 structural haplotypes. **Table S2.** Count and proportion of C4 alleles in the ABCD cohort (1,694 AFR; 5,829 EUR; 2,115 Latinx). **Table S3.** Summary of PheWAS results in the ABCD cohort. **Table S4.** Summary of 1-year follow-up PheWAS in the ABCD cohort. **Table S5.** Summary of 2-year follow-up PheWAS in the ABCD cohort. **Table S6.** Summary of C4 GREx associations with regional brain surface area in the ABCD cohort. **Table S7.** Summary of C4 GREx associations with whole brain volume (WBV), mean surface area (SA), and mean cortical thickness (CT). **Table S8.** Summary of C4 GREx associations with regional brain cortical thickness in the ABCD cohort. **Table S9.** Summary of C4 GREx associations with subcortical brain volume in the ABCD cohort. **Table S10.** Summary of Wilcoxon Rank Sum Tests comparing PQ-B symptoms and PQ-B severity at 1- and 2-year follow-up timepoints in youth in the top/bottom quartile for entorhinal cortex surface area at baseline. **Table S11.** Summary of schizophrenia PRS associations in youth of EUR ancestry. **Table S12.** Summary of schizophrenia PRS associations with regional cortical thickness in youth of EUR ancestry. **Table S13.** Summary of schizophrenia PRS associations with regional surface area in youth of EUR ancestry. **Table S14.** Summary of schizophrenia PRS associations with subcortical volume in youth of EUR ancestry.

**Additional file 2: Fig. S1.** Relationship between predicted *C4A/C4B* expression and observed *C4A/C4B* expression across ancestries. **Fig. S2.** C4 descriptives by ancestry. **Fig. S3.** Phenome-wide association between predicted C4A gene expression and behavioral phenotypes in youth of AFR ancestry. **Fig. S4.** Phenome-wide association between predicted C4A gene expression and behavioral phenotypes in youth of Latinx ancestry. **Fig. S5.** Phenome-wide association between predicted C4A gene expression and behavioral phenotypes in youth of EUR ancestry. **Fig. S6.** Sex-differences in the association between predicted C4A gene expression and behavioral phenotypes in the ABCD cohort. **Fig. S7.** Phenome-wide association between predicted C4A gene expression and behavioral phenotypes in female youth. **Fig. S8**. Phenome-wide association between predicted C4A gene expression and behavioral phenotypes in male youth. **Fig. S9.** Phenome-wide association between predicted C4A gene expression and behavioral phenotypes in the ABCD cohort at the 1-year follow-up. **Fig. S10.** Phenome-wide association between predicted C4A gene expression and behavioral phenotypes in female youth at the 1-year follow-up. **Fig. S11.** Phenome-wide association between predicted C4A gene expression and behavioral phenotypes in male youth at the 1-year follow-up. **Fig. S12.** Phenome-wide association between predicted *C4A* gene expression and behavioral phenotypes in the ABCD cohort at the 2-year follow-up. **Fig. S13.** Phenome-wide association between predicted *C4A* gene expression and behavioral phenotypes in female youth at the 2-year follow-up. **Fig. S14.** Phenome-wide association between predicted *C4A* gene expression and behavioral phenotypes in male youth at the 2-year follow-up. **Fig. S15.** Relationship between C4 haplotypes and baseline psychosis-like experiences in the multi-ancestry ABCD sample. **Fig. S16.** Factors influencing entorhinal cortex surface area at baseline.

**Additional file 3.** Review history.

## Data Availability

The datasets supporting the conclusions of this article are available through the Adolescent Brain Cognitive Development study [21] on the National Data Archive (10.15154/1519007) and with application to the UK Biobank (https://www.ukbiobank.ac.uk/) [35]. Genotype array and RNA sequencing data were obtained from PsychENCODE (https:// doi.org/10.7303/syn12080241) [74]. The codes used to perform statistical analyses are available under the MIT license on GitHub (https://github.com/leamhernandez/C4A-ABCD) [75] and have also been deposited in Zenodo (10.5281/zenodo.7542574) [76].
