# Supplementary material for: Multi-ancestry phenome-wide association of complement component 4 variation with psychiatric and brain phenotypes in youth
Source: Genome Biol. 2023 Mar 7;24:42. doi: 10.1186/s13059-023-02878-0 (PMC9990244; doi:10.1186/s13059-023-02878-0)
Supplement: Supplementary file 2 — Additional file 2: Fig. S1. Relationship between predicted C4A/C4B expression and observed C4A/C4B expression across ancestries. Fig. S2. C4 descriptives by ancestry. Fig. S3. Phenome-wide association between predicted C4A gene expression and behavioral phenotypes in youth of AFR ancestry. Fig. S4. Phenome-wide association between predicted C4A gene expression and behavioral phenotypes in youth of Latinx ancestry. Fig. S5. Phenome-wide association between predicted C4A gene expression and behavioral phenotypes in youth of EUR ancestry. Fig. S6. Sex-differences in the association between predicted C4A gene expression and behavioral phenotypes in the ABCD cohort. Fig. S7. Phenome-wide association between predicted C4A gene expression and behavioral phenotypes in female youth. Fig. S8. Phenome-wide association between predicted C4A gene expression and behavioral phenotypes in male youth. Fig. S9. Phenome-wide association between predicted C4A gene expression and behavioral phenotypes in the ABCD cohort at the 1-year follow-up. Fig. S10. Phenome-wide association between predicted C4A gene expression and behavioral phenotypes in female youth at the 1-year follow-up. Fig. S11. Phenome-wide association between predicted C4A gene expression and behavioral phenotypes in male youth at the 1-year follow-up. Fig. S12. Phenome-wide association between predicted C4A gene expression and behavioral phenotypes in the ABCD cohort at the 2-year follow-up. Fig. S13. Phenome-wide association between predicted C4A gene expression and behavioral phenotypes in female youth at the 2-year follow-up. Fig. S14. Phenome-wide association between predicted C4A gene expression and behavioral phenotypes in male youth at the 2-year follow-up. Fig. S15. Relationship between C4 haplotypes and baseline psychosis-like experiences in the multi-ancestry ABCD sample. Fig. S16. Factors influencing entorhinal cortex surface area at baseline. [file 13059_2023_2878_MOESM2_ESM.docx]

# **Additional File 1**

# **Multi-ancestry phenome-wide association of complement component 4 variation with psychiatric and brain phenotypes in youth**

# Leanna M. Hernandez, PhD, Minsoo Kim, BS, Pan Zhang, PhD, Richard A.I. Bethlehem, PhD, Gil Hoftman, MD, PhD, Robert Loughnan, BS, Diana Smith, BS, Susan Y. Bookheimer, PhD, Chun Chieh Fan, PhD, Carrie E. Bearden, PhD, Wesley K. Thompson, PhD^5^ Michael J. Gandal, MD, PhD

**Fig. S1.** Relationship between predicted *C4A/C4B* expression and observed *C4A/C4B* expression across ancestries.

**Fig. S2.** C4 descriptives by ancestry.

**Fig. S3.** Phenome-wide association between predicted C4A gene expression and behavioral phenotypes in youth of AFR ancestry.

**Fig. S4.** Phenome-wide association between predicted C4A gene expression and behavioral phenotypes in youth of Latinx ancestry.

**Fig. S5.** Phenome-wide association between predicted C4A gene expression and behavioral phenotypes in youth of EUR ancestry.

**Fig. S6.** Sex-differences in the association between predicted C4A gene expression and behavioral phenotypes in the ABCD cohort.

**Fig. S7.** Phenome-wide association between predicted C4A gene expression and behavioral phenotypes in female youth.

**Fig. S8**. Phenome-wide association between predicted C4A gene expression and behavioral phenotypes in male youth.

**Fig. S9.** Phenome-wide association between predicted C4A gene expression and behavioral phenotypes in the ABCD cohort at the 1-year follow-up.

**Fig. S10.** Phenome-wide association between predicted C4A gene expression and behavioral phenotypes in female youth at the 1-year follow-up.

**Fig. S11.** Phenome-wide association between predicted C4A gene expression and behavioral phenotypes in male youth at the 1-year follow-up.

**Fig. S12.** Phenome-wide association between predicted *C4A* gene expression and behavioral phenotypes in the ABCD cohort at the 2-year follow-up.

**Fig. S13.** Phenome-wide association between predicted *C4A* gene expression and behavioral phenotypes in female youth at the 2-year follow-up.

**Fig. S14.** Phenome-wide association between predicted *C4A* gene expression and behavioral phenotypes in male youth at the 2-year follow-up.

**Fig. S15.** Relationship between C4 haplotypes and baseline psychosis-like experiences in the multi-ancestry ABCD sample.

**Fig. S16.** Factors influencing entorhinal cortex surface area at baseline.

**Fig. S1. Relationship between predicted C4A/C4B expression and observed C4A/C4B expression**. A) Predicted C4A/C4B expression was significantly associated with measured brain expression in 340 AFR, 50 Latinx, and 863 European samples from the PsychENCODE dataset. B) Measured C4A/C4B gene expression was strongly associated with corresponding predicted gene expression in 23 European samples ages 5-15 from the PsychENCODE dataset.


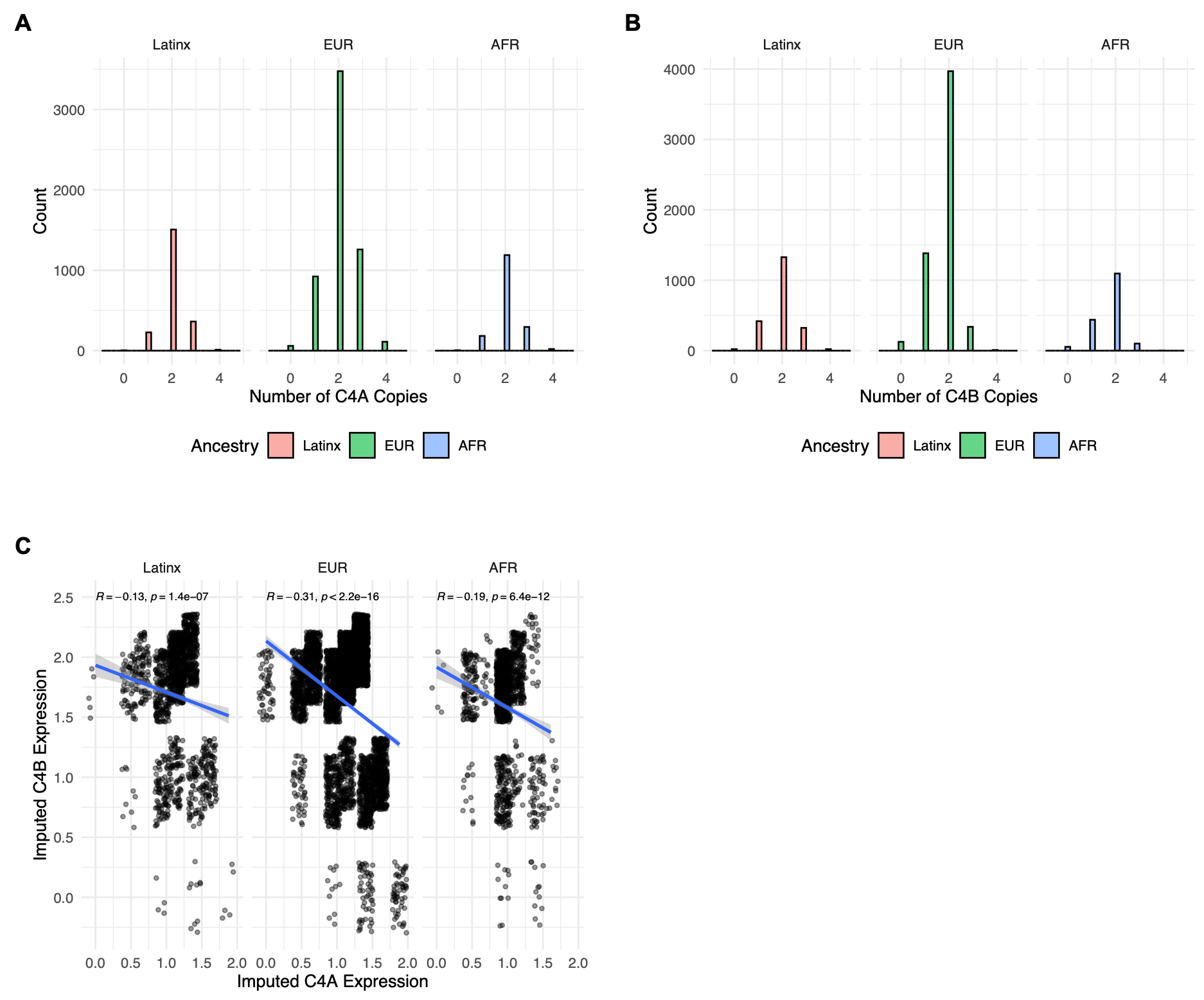


**Fig. S2. *C4* descriptives by ancestry.** A) Histograms showing the number of *C4A* copies by ancestry group. B) Histograms showing the number of *C4B* copies by ancestry group. C) Spearman rank order correlations between genetically imputed *C4A* and *C4B* brain expression.


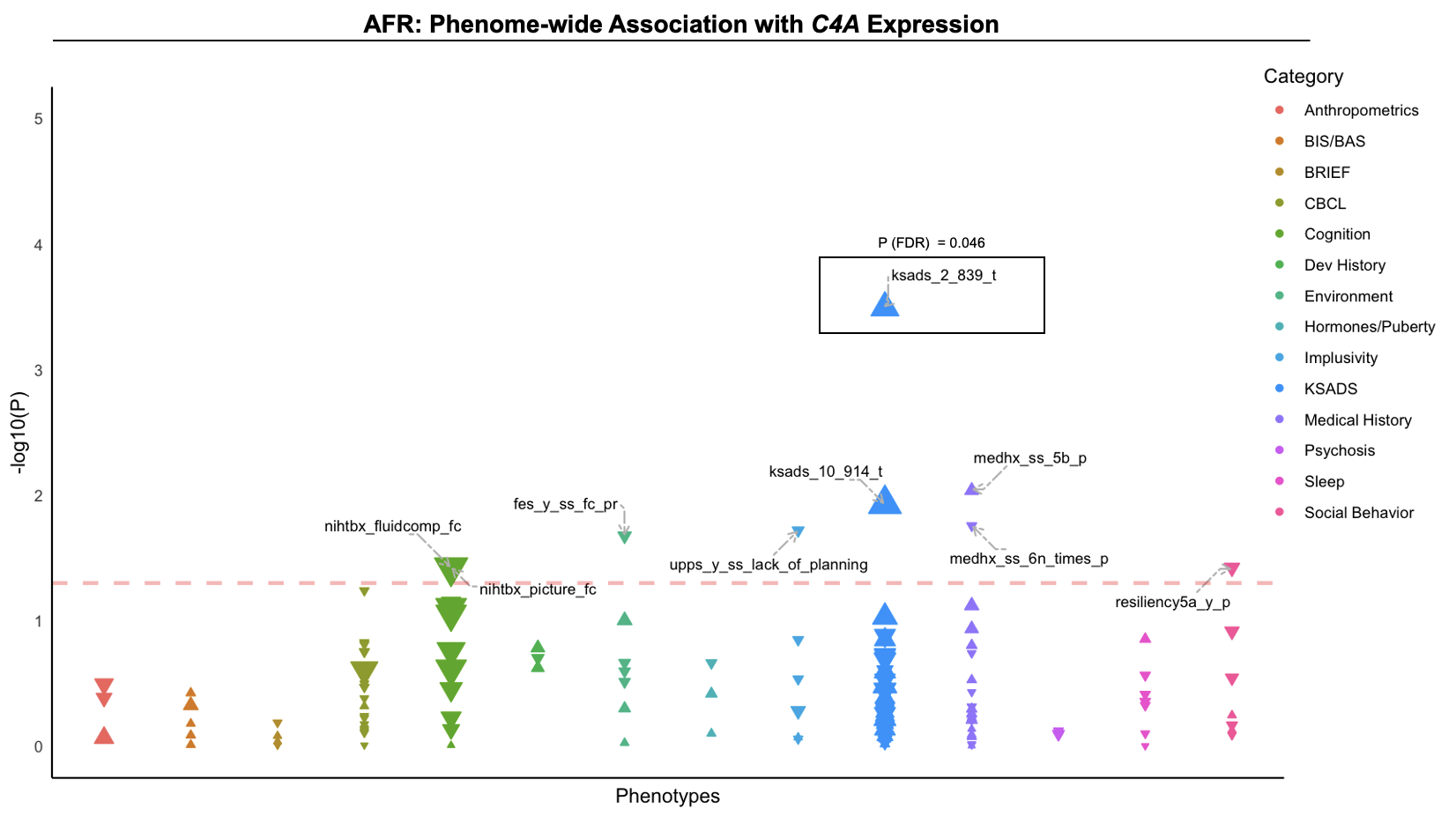


**Fig. S3. Phenome-wide association between predicted *C4A* gene expression and behavioral phenotypes in youth of AFR ancestry.** Phenotypes are grouped into broad categories by color. The magnitude of effect (i.e., absolute 𝛽) is represented by size of the data points. Negative or positive associations with genetically predicted *C4A* expression are indicated by the direction of arrows. The threshold for P < 0.05 is indicated by a horizontal red line; labeled points indicate nominally significant associations (P < 0.05).


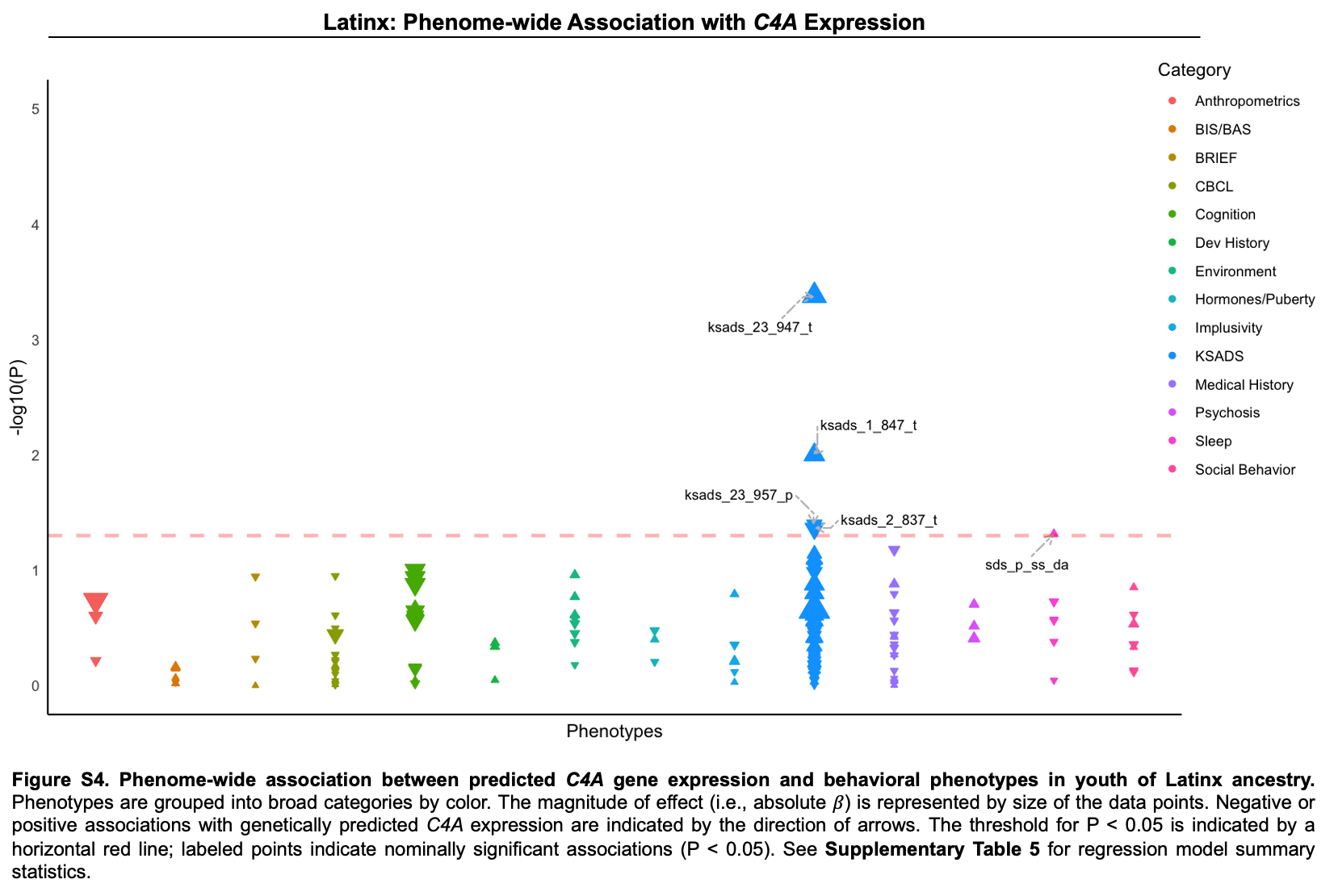


**Fig. S4. Phenome-wide association between predicted *C4A* gene expression and behavioral phenotypes in youth of Latinx ancestry.** Phenotypes are grouped into broad categories by color. The magnitude of effect (i.e., absolute 𝛽) is represented by size of the data points. Negative or positive associations with genetically predicted *C4A* expression are indicated by the direction of arrows. The threshold for P < 0.05 is indicated by a horizontal red line; labeled points indicate nominally significant associations (P < 0.05).


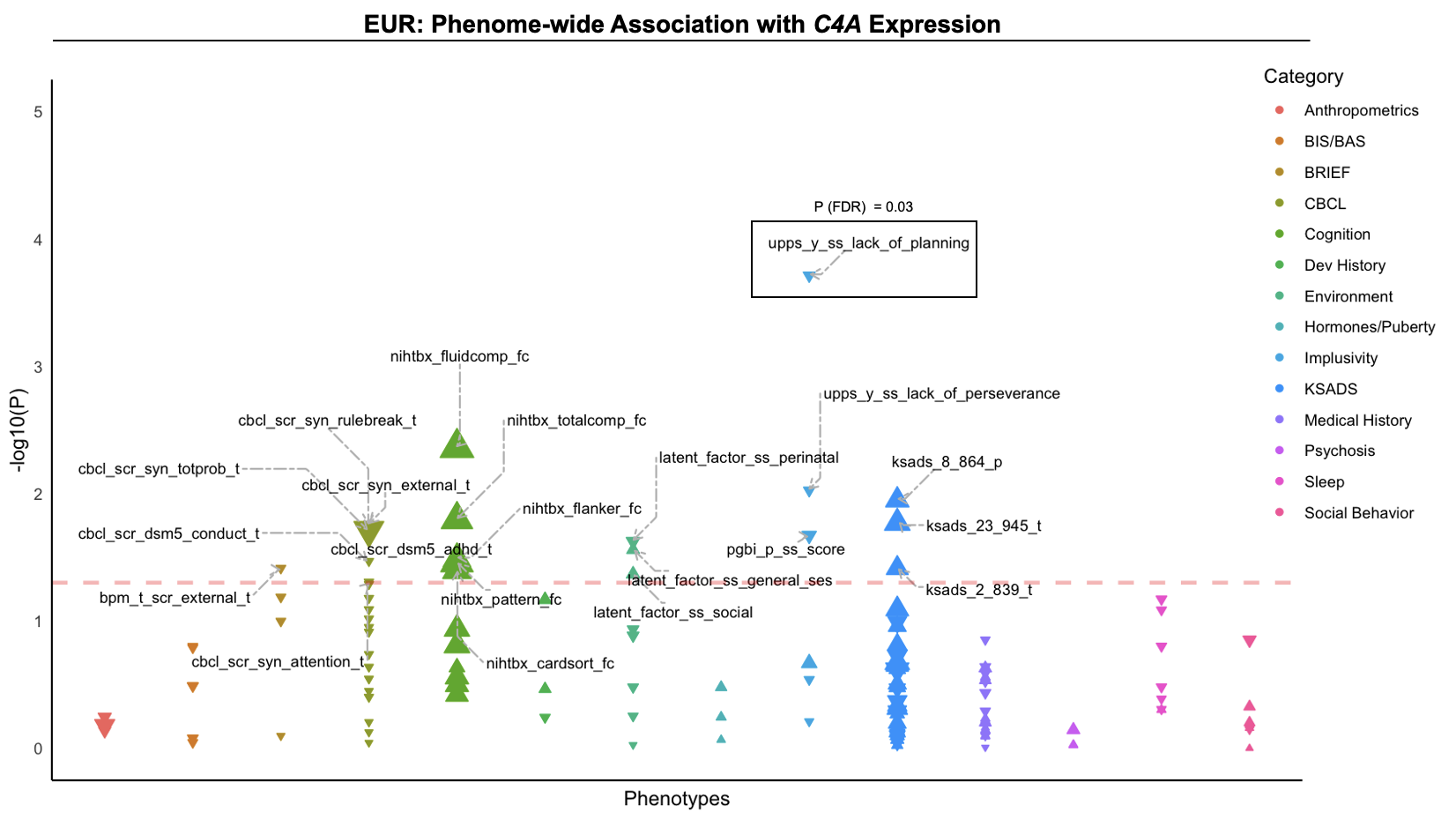


**Fig. S5. Phenome-wide association between predicted *C4A* gene expression and behavioral phenotypes in youth of EUR ancestry.** Phenotypes are grouped into broad categories by color. The magnitude of effect (i.e., absolute 𝛽) is represented by size of the data points. Negative or positive associations with genetically predicted *C4A* expression are indicated by the direction of arrows. The threshold for P < 0.05 is indicated by a horizontal red line; labeled points indicate nominally significant associations (P < 0.05).


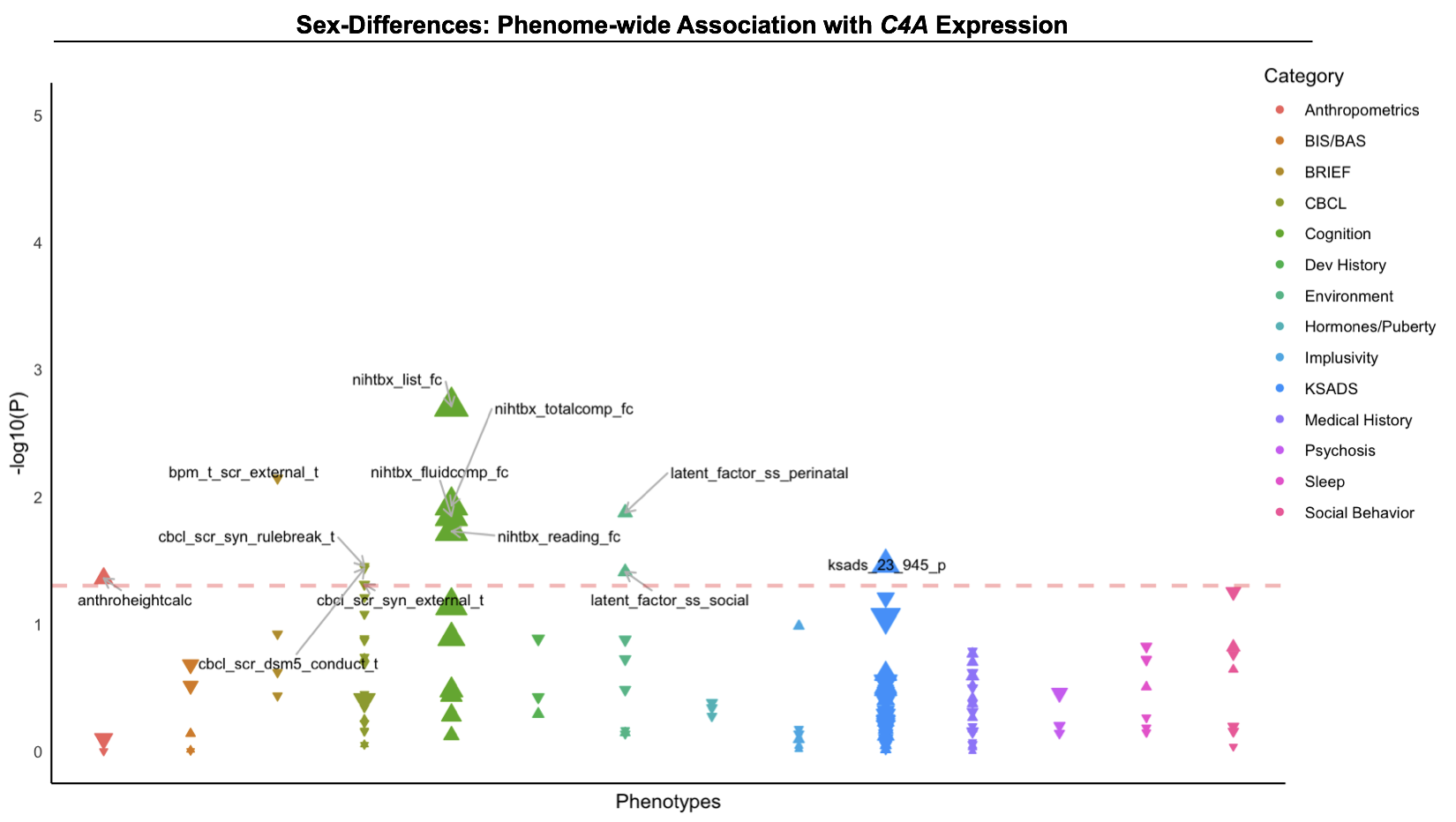


**Fig. S6. Sex-differences in the association between predicted *C4A* gene expression and behavioral phenotypes in the ABCD cohort.** Phenotypes are grouped into broad categories by color. The magnitude of effect (i.e., absolute 𝛽) is represented by size of the data points. Negative or positive associations with genetically predicted *C4A* expression are indicated by the direction of arrows. The threshold for P < 0.05 is indicated by a horizontal red line; labeled points indicate nominally significant associations (P < 0.05).


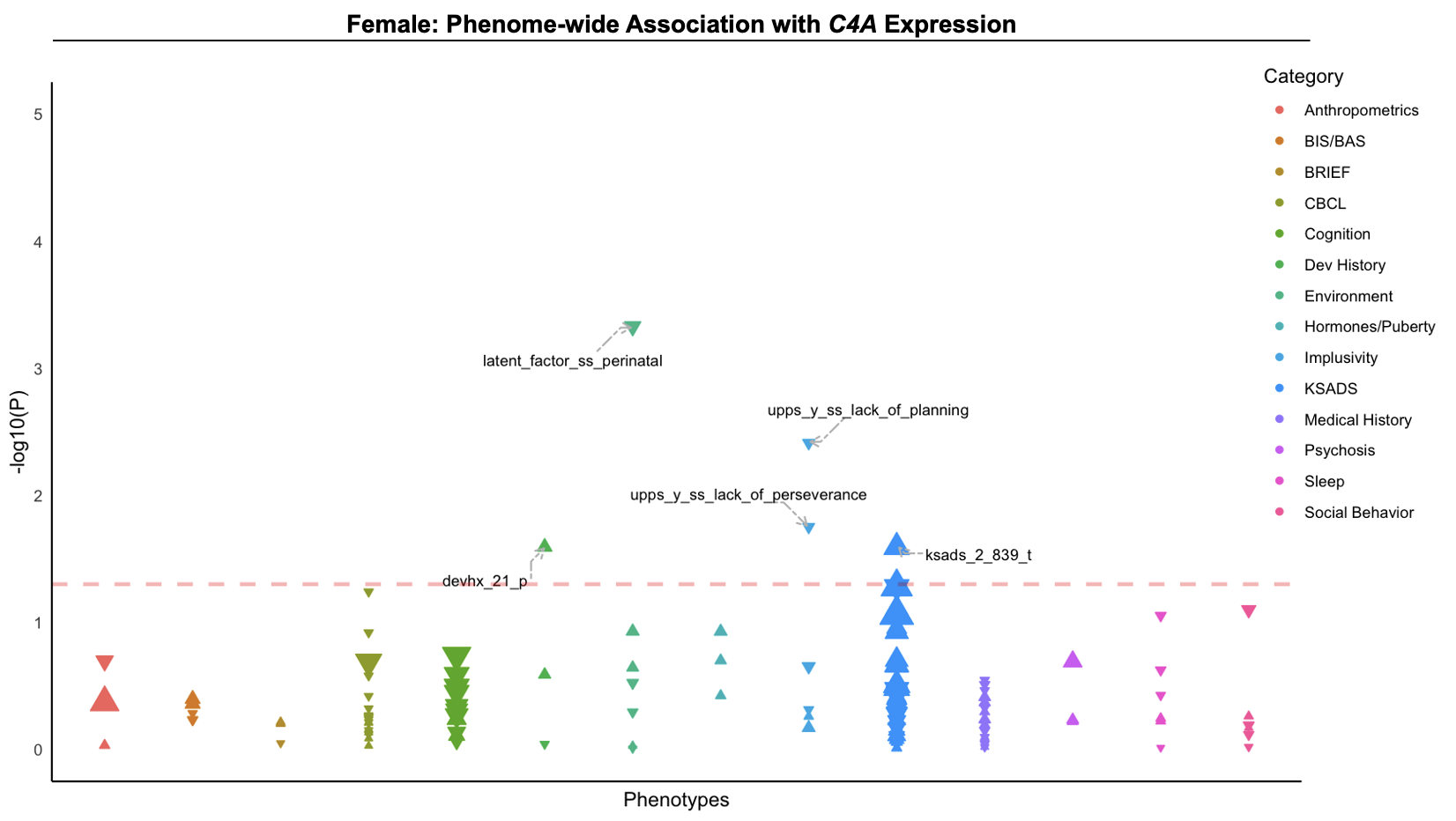


**Fig. S7. Phenome-wide association between predicted *C4A* gene expression and behavioral phenotypes in female youth.** Phenotypes are grouped into broad categories by color. The magnitude of effect (i.e., absolute 𝛽) is represented by size of the data points. Negative or positive associations with genetically predicted *C4A* expression are indicated by the direction of arrows. The threshold for P < 0.05 is indicated by a horizontal red line; labeled points indicate nominally significant associations (P < 0.05).


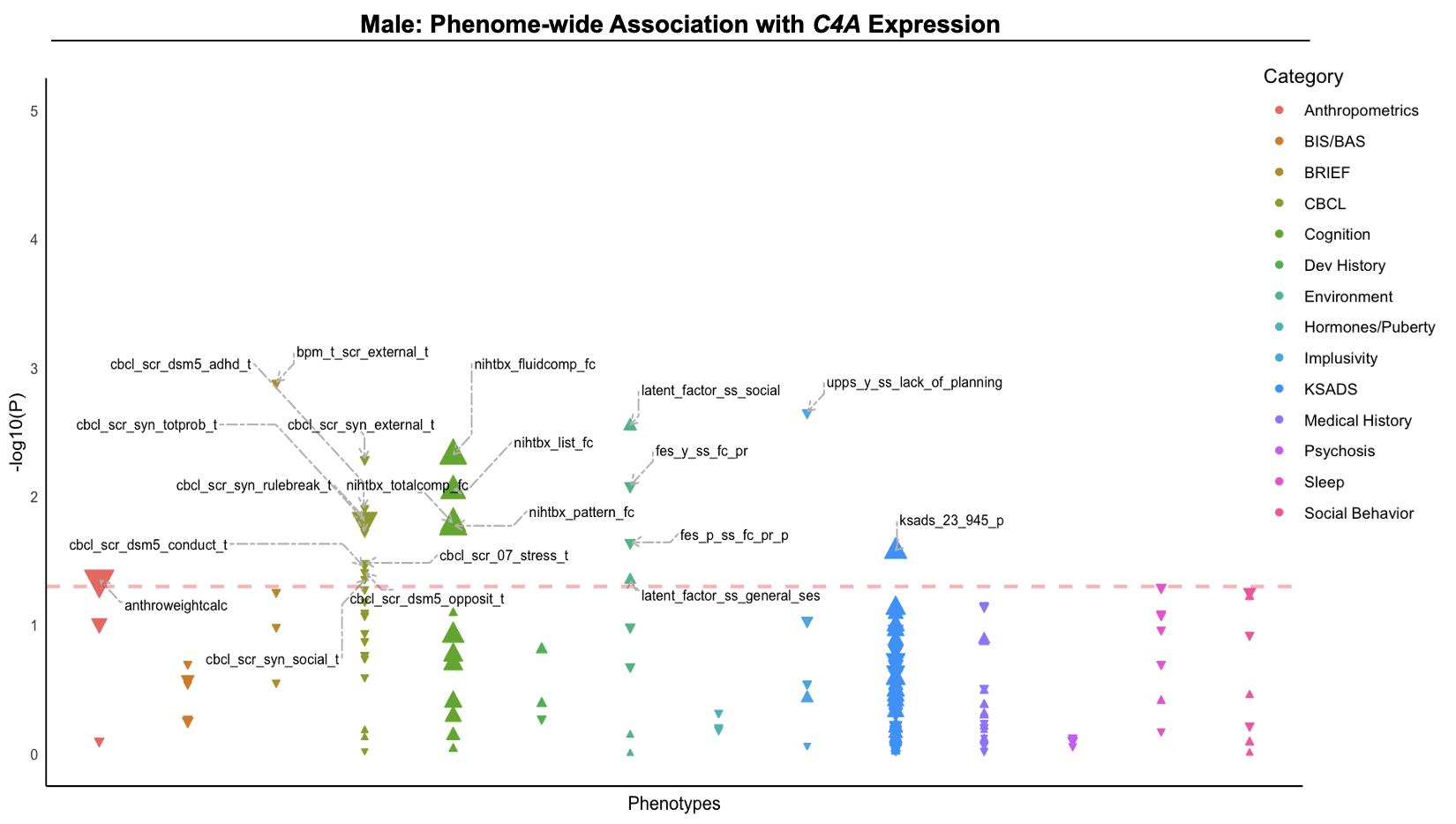


**Fig. S8. Phenome-wide association between predicted *C4A* gene expression and behavioral phenotypes in male youth.** Phenotypes are grouped into broad categories by color. The magnitude of effect (i.e., absolute 𝛽) is represented by size of the data points. Negative or positive associations with genetically predicted *C4A* expression are indicated by the direction of arrows. The threshold for P < 0.05 is indicated by a horizontal red line; labeled points indicate nominally significant associations (P < 0.05).


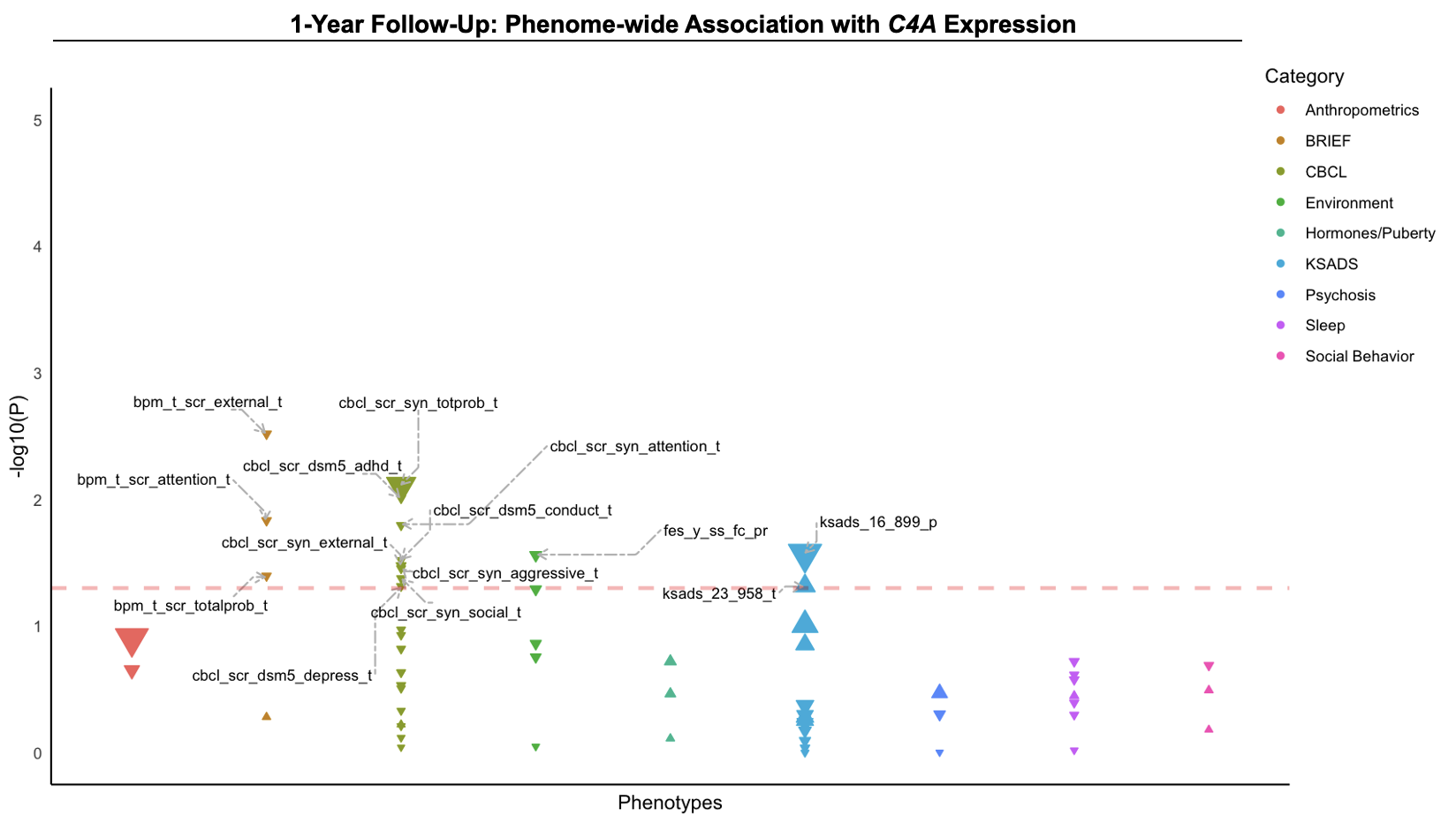


**Fig. S9. Phenome-wide association between predicted *C4A* gene expression and behavioral phenotypes in the ABCD cohort at the 1-year follow-up.** Phenotypes are grouped into broad categories by color. The magnitude of effect (i.e., absolute 𝛽) is represented by size of the data points. Negative or positive associations with genetically predicted *C4A* expression are indicated by the direction of arrows. The threshold for P < 0.05 is indicated by a horizontal red line; labeled points indicate nominally significant associations (P < 0.05).


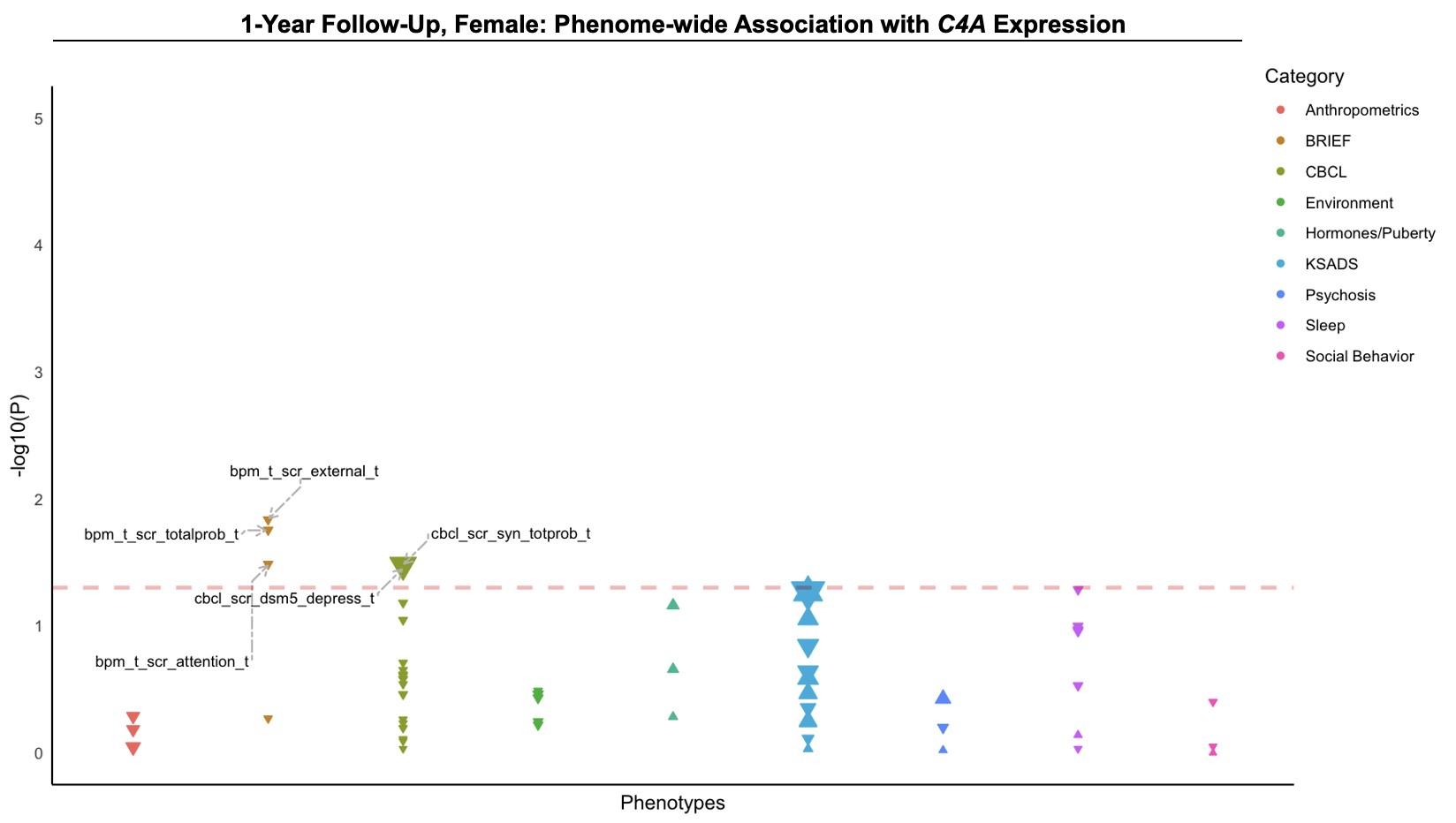


**Fig. S10. Phenome-wide association between predicted *C4A* gene expression and behavioral phenotypes in female youth at the 1-year follow-up.** Phenotypes are grouped into broad categories by color. The magnitude of effect (i.e., absolute 𝛽) is represented by size of the data points. Negative or positive associations with genetically predicted *C4A* expression are indicated by the direction of arrows. The threshold for P < 0.05 is indicated by a horizontal red line; labeled points indicate nominally significant associations (P < 0.05).


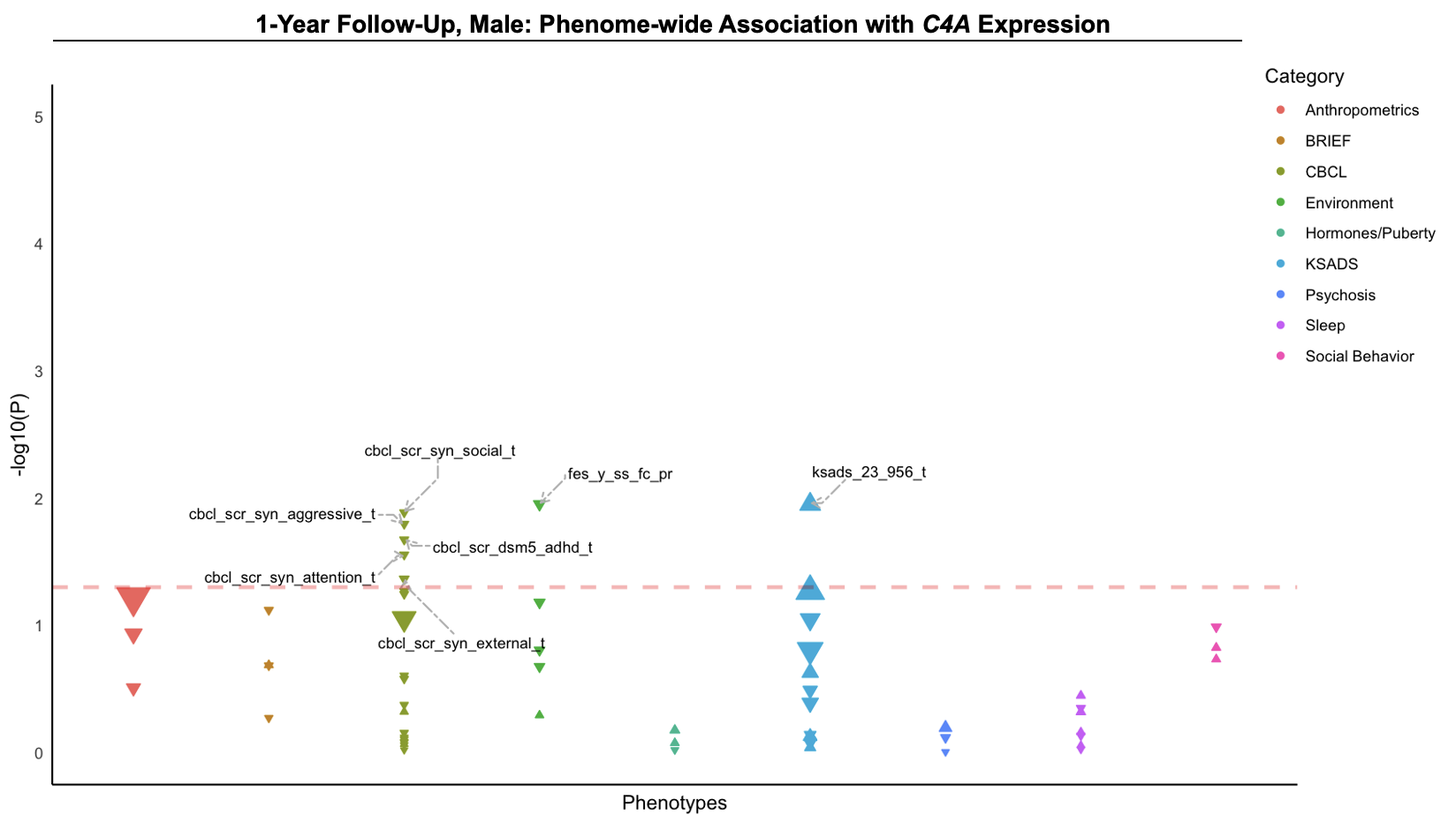


**Fig. S11. Phenome-wide association between predicted *C4A* gene expression and behavioral phenotypes in male youth at the 1-year follow-up.** Phenotypes are grouped into broad categories by color. The magnitude of effect (i.e., absolute 𝛽) is represented by size of the data points. Negative or positive associations with genetically predicted *C4A* expression are indicated by the direction of arrows. The threshold for P < 0.05 is indicated by a horizontal red line; labeled points indicate nominally significant associations (P < 0.05).


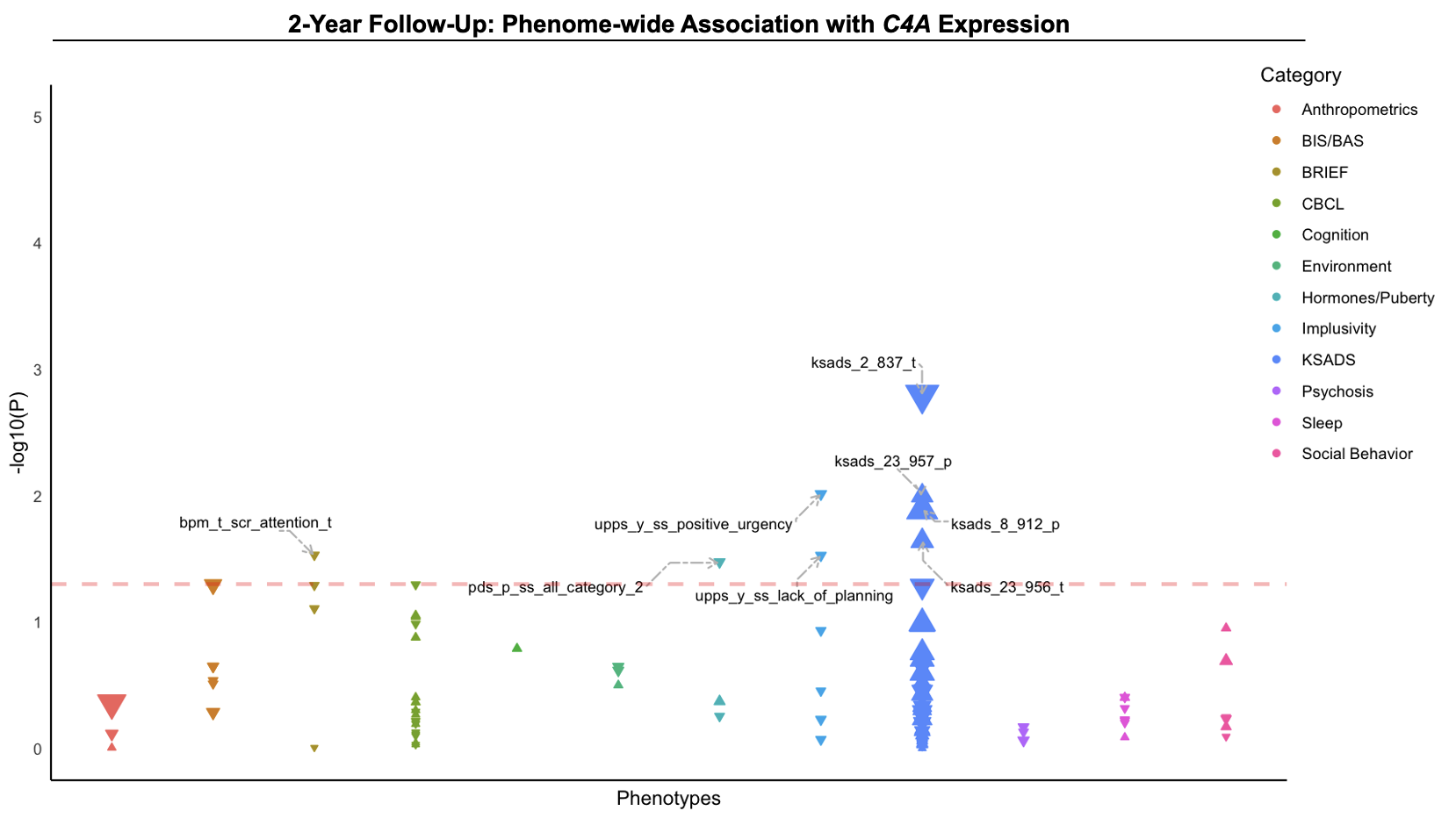


**Fig. S12. Phenome-wide association between predicted *C4A* gene expression and behavioral phenotypes in the ABCD cohort at the 2-year follow-up.** Phenotypes are grouped into broad categories by color. The magnitude of effect (i.e., absolute 𝛽) is represented by size of the data points. Negative or positive associations with genetically predicted *C4A* expression are indicated by the direction of arrows. The threshold for P < 0.05 is indicated by a horizontal red line; labeled points indicate nominally significant associations (P < 0.05).


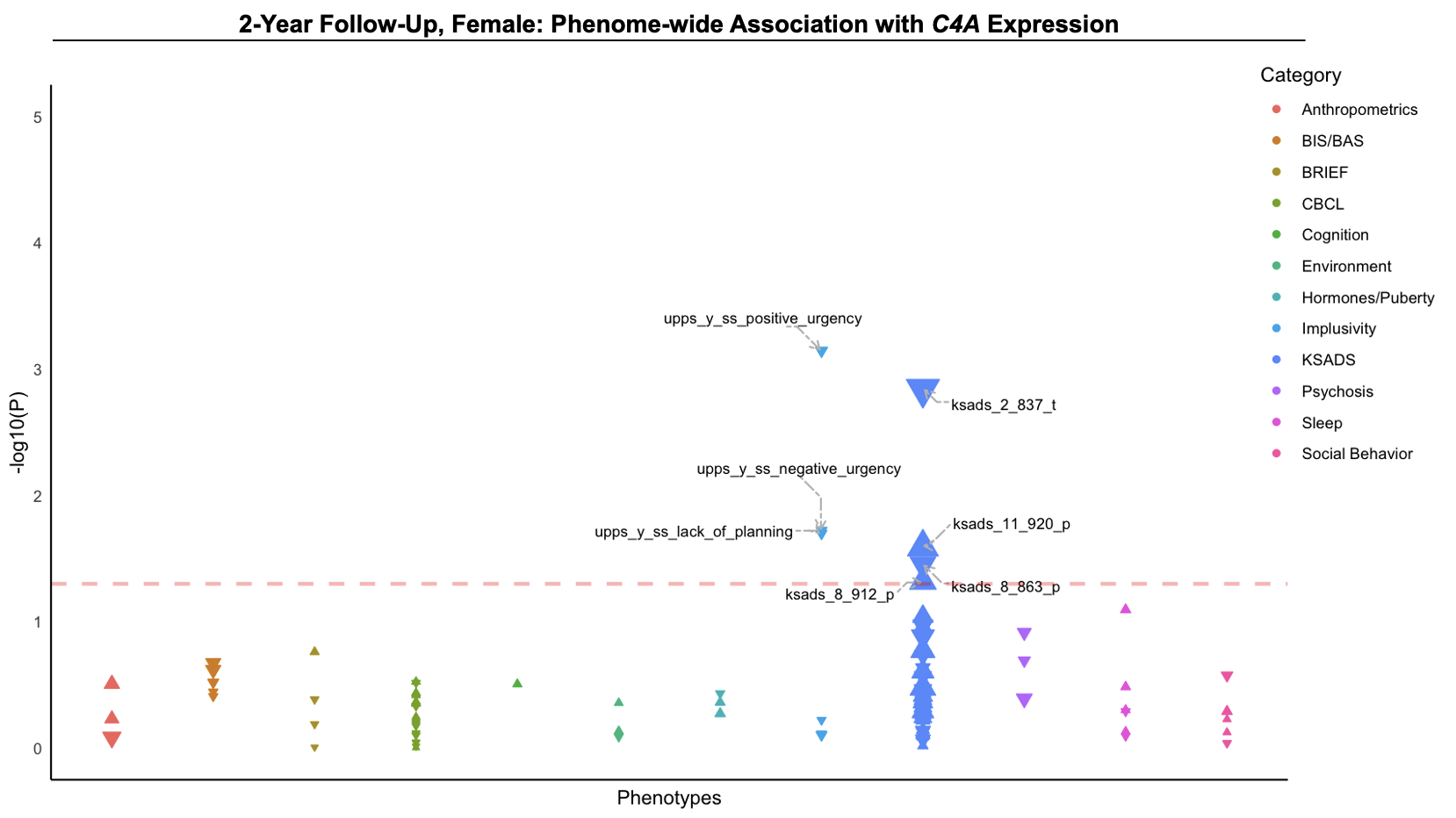


**Fig. S13. Phenome-wide association between predicted *C4A* gene expression and behavioral phenotypes in female youth at the 2-year follow-up.** Phenotypes are grouped into broad categories by color. The magnitude of effect (i.e., absolute 𝛽) is represented by size of the data points. Negative or positive associations with genetically predicted *C4A* expression are indicated by the direction of arrows. The threshold for P < 0.05 is indicated by a horizontal red line; labeled points indicate nominally significant associations (P < 0.05).


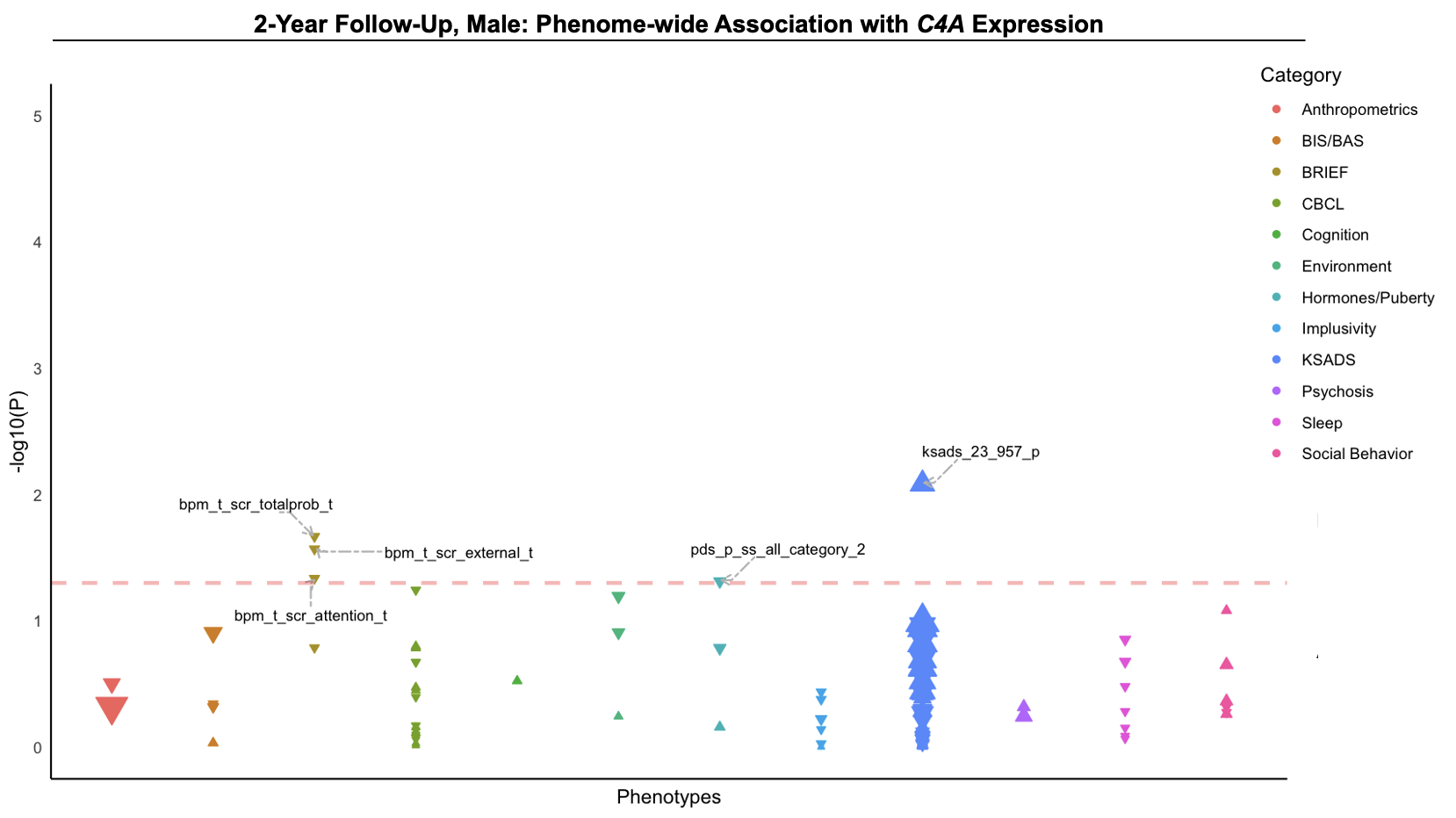


**Fig. S14. Phenome-wide association between predicted *C4A* gene expression and behavioral phenotypes in male youth at the 2-year follow-up.** Phenotypes are grouped into broad categories by color. The magnitude of effect (i.e., absolute 𝛽) is represented by size of the data points. Negative or positive associations with genetically predicted *C4A* expression are indicated by the direction of arrows. The threshold for P < 0.05 is indicated by a horizontal red line; labeled points indicate nominally significant associations (P < 0.05).

**Fig. S15. Relationship between C4 haplotypes and baseline psychosis-like experiences in the multi-ancestry ABCD sample.** Regression analyses were performed for the joint model assessing the impact of 14 unique allelic combinations on psychosis-like experiences, setting the BS:BS haplotype as reference. The AL:AL haplotype was nominally associated with PQ-B_sym_, PQ-B_sev_, and case/control status (P < 0.05). Associations were no longer significant after Bonferroni correction for multiple testing. *P < 0.05

**Fig. S16. Factors influencing entorhinal cortex surface area at baseline.** We characterized the effect of relevant anthropometric and behavioral phenotypes on entorhinal cortex surface are at baseline. In addition to *C4A* GREx, standing height, crystalized cognition, socioeconomic status, weight, total cognition, waist circumference, and weekend screen time showed significant associations with entorhinal cortex surface area.
