# Supplementary material for: Multi-ancestry phenome-wide association of complement component 4 variation with psychiatric and brain phenotypes in youth
Source: Genome Biol. 2023 Mar 7;24:42. doi: 10.1186/s13059-023-02878-0 (PMC9990244; doi:10.1186/s13059-023-02878-0)
Supplement: Supplementary file 3 — Additional file 3. Review history. [file 13059_2023_2878_MOESM3_ESM.docx]

Review History

**First round of review**

**Reviewer 1**

**Were you able to assess all statistics in the manuscript, including the appropriateness of statistical tests used?**

No, I do not feel adequately qualified to assess the statistics.

**Comments to author:**

Hernandez et al have produced a comprehensive functional investigation of a very important locus, C4A, in terms of genetic liability for schizophrenia, which is a neurodevelopmental disorder. It is the study of the relationship between genetically-regulated expression (GREx) of C4A and childhood brain structure, cognition, and psychiatric symptoms. The authors find that GREx of C4A is associated with reduced entorhinal cortex surface area at 9-10 years-of-age, which predicts greater number and severity of psychosis like events 1- and 2- years later.

This is a very impressive study that is well written and presented, and is relatively easy to follow. The methods are appropriate to the aims of the study, are well described, and the necessary controls are included. The conclusions are adequately supported by the data shown. Sufficient details are provided to allow replication and comparison with related analyses that may have been performed. The work represents a significant advance over previously published studies in that it is the first study of GREx of C4A in a childhood sample. The paper will be of broad interest to others in the field of psychiatric genetics.

I have one major issue and two minor issues.

Major

1. C4 structural haplotypes (imputed using GWAS data) were used to calculate GREx for C4A and C4B using previously described weights (Sekar et al, 2016). Sekar and colleagues assessed how C4 structural variation related to RNA expression of C4A and C4B in eight panels of post-mortem human adult brain samples. How do the authors know that a similar genotype-phenotype relationship exists between C4 structural haplotypes and C4A expression in the brains of children, as these are the sample type being used here?

Minor

1. From Results, "In adults and schizophrenia patients, genetic variation within the C4 locus has been associated with poorer cognition and memory (20,25-27)." Are references 25-27 correct as they are studies of the CSMD1, CSMD2 and CFH genes and not C4?

2. Figure 3. Legend for panels C) and D) are in fact for D) and E), and the legend for C) is missing.

**Reviewer 2**

**Were you able to assess all statistics in the manuscript, including the appropriateness of statistical tests used?**

Yes, and I have assessed the statistics in my report.

**Comments to author:**

Hernandez and colleagues conducted an interesting study to investigate the effect of genetically-predicted C4 expression on psychiatric symptoms and traits. The analyses are well designed and the methods are appropriate for the aims of the study. Nevertheless, I have some comments that the authors should consider in their revision.

1. The authors refer to their study as "multi-ethnic". However, since they are analyzing genomic data, the analyses were correctly conducted with respect to genetically-defined ancestry groups. Accordingly, it would be more appropriate to refer to the study as "multi-ancestry". The term "ethnic" can have different meanings depending on the socio-cultural context.

2. It is not clear whether the weights to predict genetically-regulated C4 expression were derived from ancestry groups or whether they were derived from individuals of European descent. This should be clearly reported. If European-descent weights were used across the ancestry groups, this should be reported as a limitation.

3. It would be better to report the power analysis at the beginning of the results to show that the null results reported are not due to a lack of power.

4. It would be interesting to investigate whether there are differences in the C4 associations among individuals with high vs. low non-MHC SCZ PRS with respect to psychotic-like experiences and childhood cognition.

5. Although the study is focused on genetically-regulated C4 expression, it would be interesting to include information regarding the association of C4 haplotypes with the phenotypes investigated. This would permit the assessment of the convergence between transcriptomic and genetic associations.

6. The authors should discuss their findings with respect to previous studies that investigated C4 with respect to psychiatric disorders other than SCZ (e.g., PMID: 31570195).

**Reviewer 3**

**Were you able to assess all statistics in the manuscript, including the appropriateness of statistical tests used?**

Yes, and I have assessed the statistics in my report.

**Comments to author:**

In this study, Hernandez et al. use models of genetically regulated expression of C4 to study its relationship to youth neuropsychiatric phenotypes, as well as structural MRI derived brain indices. This study is rigorously performed and the finding of an association with the surface area of entorhinal cortex is interesting, however, I have a few suggestions that I feel would improve the manuscript.

Major Comments:

1. Page 10: The authors found evidence that youth in the bottom quartile of entorhinal SA were more likely to report psychotic-like experiences at 1-year follow up. I think the authors should also test and report the results of using entorhinal surface are as a continuous predictor, given that comparing the top and bottom quartiles will be a more exaggerated effect.

2. Do the authors have a sense how much the C4 variation is captured by GREx?

3. Page 10: At the 2-year follow-up there is no mention of the effect in males, was there a difference in psychotic-like experiences as seen in females at this time-point?

4. Given that the C4 GREx was not associated with psychotic-like experiences by itself, I think that the authors should be more mindful of the fact that there will be many other factors that will influence entorhinal SA, and thus, be careful as to not overstate the relationship between C4 and subsequent PLEs. As a result, I suggest that the authors should report the variance explained in entorhinal SA by the C4 GREx and compare that to other relevant phenotypes collected in this cohort. This would allow the reader to contextualise the strength of the effect of C4 related variation versus other factors.

5. Related to the above, is there is a reason that the authors only tested whether the entorhinal SA was associated with PLEs? One could probably also justify performing the same pheWAS of entorhinal SA, which naturally would have a higher multiple-testing burden.

6. It would be interesting to see if entorhinal surface area partly mediates the association of C4 GREx with the lack of planning subscale for which C4 demonstrated an association after multiple-testing correction in the pheWAS.

7. Perhaps the authors could consider testing whether there is an interaction with a cortical surface area polygenic score with C4, rather than just the schizophrenia PRS.

8. The discussion would benefit from more discussion about the neuropathology/neurobiology of the entorhinal cortex.

9. There should be more discussion of the limitations of this study, I feel this is a bit lacking.

10. I also think that the authors overstate the utility of this finding in the concluding statement "Overall, these data provide a mechanistic, genetic basis for the identification of brain-based biomarkers predictive of psychosis risk"

Minor Comments:

1. Line 14 - the authors refer to the MHC as the strongest schizophrenia association. The authors should specify that this is the most statistically significant association, there are larger effect size rare and structural variants (e.g. SETD1A, 22q11.2 deletion).

2. The authors should explain more how the C4 GREx are calculated in this manuscript. The methods for this are hidden in the supplementary of the Sekar et al. paper and I think it's important to emphasise to the reader that this is not a GREx constructed of single SNPs like in conventional TWAS but is based on C4 structure.

**Authors Response**

**Point-by-point responses to the reviewers’ comments:**

**Reviewer 1:**

***Hernandez et al have produced a comprehensive functional investigation of a very important locus, C4A, in terms of genetic liability for schizophrenia, which is a neurodevelopmental disorder. It is the study of the relationship between genetically-regulated expression (GREx) of C4A and childhood brain structure, cognition, and psychiatric symptoms. The authors find that GREx of C4A is associated with reduced entorhinal cortex surface area at 9-10 years-of-age, which predicts greater number and severity of psychosis like events 1- and 2- years later.***

***This is a very impressive study that is well written and presented, and is relatively easy to follow. The methods are appropriate to the aims of the study, are well described, and the necessary controls are included. The conclusions are adequately supported by the data shown. Sufficient details are provided to allow replication and comparison with related analyses that may have been performed. The work represents a significant advance over previously published studies in that it is the first study of GREx of C4A in a childhood sample. The paper will be of broad interest to others in the field of psychiatric genetics.***

We thank the reviewer for their comments and feedback on the advance presented with our manuscript. We are glad the reviewer found the work well presented and easy to follow.

***I have one major issue and two minor issues.***

***Major***

***1. C4 structural haplotypes (imputed using GWAS data) were used to calculate GREx for C4A and C4B using previously described weights (Sekar et al, 2016). Sekar and colleagues assessed how C4 structural variation related to RNA expression of C4A and C4B in eight panels of post-mortem human adult brain samples. How do the authors know that a similar genotype-phenotype relationship exists between C4 structural haplotypes and C4A expression in the brains of children, as these are the sample type being used here?***

We thank the reviewer for bringing up this important point regarding the transferability of adult-derived expression weights to childhood samples. Indeed, the postmortem human brain tissue RNA samples from which the *C4A* and *C4B* imputation weights were derived consist of donors between the ages of 19-68 (Sekar et al., 2016). In order to validate the accuracy of applying these adult-derived weights to impute brain gene expression in children and early adolescents, we used independent data from the PsychENCODE consortium to compare measured versus imputed *C4A* and *C4B* gene expression in a subsample of European ancestry donors between the ages of 5-15. In this subsample, we find a significant positive relationship between measured and imputed C4A (R=0.59, p=0.003) and C4B (R=0.42, p=0.046), confirming that there is a similar genotype-phenotype relationship between C4 structural haplotypes and C4 gene expression in the brains of children. We now include the results of this validation in Supplementary Figure 1B and have added the following text to the Methods section of the revised manuscript:

*“We validated the accuracy of C4 imputation weights for predicting gene expression in ancestrally diverse cohorts using data from 340 AFR, 863 EUR, and 50 Latinx donors from the PsychENCODE Consortium (Supplementary Fig. 1). We further validated that these weights were accurate in the subset of 5-15 year-old samples from PsychENCODE which match the*

*adolescent period investigated in this study.”*

***Minor
1. From Results, "In adults and schizophrenia patients, genetic variation within the C4 locus has been associated with poorer cognition and memory (20,25-27)." Are references 25-27 correct as they are studies of the CSMD1, CSMD2 and CFH genes and not C4?***

We apologize for the lack of clarity regarding the relationship between complement component 4, CSMD, and CFH genes and cognition. CSMD1 and CFH are key regulators of the complement system (Escudero-Esparza et al., FASEB J., 2013; Kennedy et al., J Immunol, 2016; Kraus et al., J Immunol, 2006; Ripoche et al., 1988; Wu et al., Nat Immunol, 2009), and CSMD1 has also implicated in the pathophysiology of schizophrenia (Havik et al., Biol Psychiatry, 2011; Ripke et al., Nat Genet, 2011Schizophrenia Working Group of the Psychiatric Genomics Consortium, Nature, 2014). We have revised the text to reflect that some of the references refer to genes that affect the functioning of the complement system.

*“In adults and schizophrenia patients, genetic variation within the C4 locus, as well as in genes that regulate the complement system, has been associated with poorer cognition and memory (20,25–27).”*

***2. Figure 3. Legend for panels C) and D) are in fact for D) and E), and the legend for C) is missing.***

We thank the reviewer for altering us to this error. The Figure legend has been corrected in the

revised manuscript. We have added the following to the Figure 3 legend:

*C) Replication of the relationship between C4A GREx and entorhinal cortex (EC) surface area in 8,357 adult females and 7,790 adult males of European ancestry from the UK Biobank.*

**Reviewer 2:**

***Hernandez and colleagues conducted an interesting study to investigate the effect of genetically-predicted C4 expression on psychiatric symptoms and traits. The analyses are well designed and the methods are appropriate for the aims of the study. Nevertheless, I have some comments that the authors should consider in their revision.***

***1. The authors refer to their study as "multi-ethnic". However, since they are analyzing genomic data, the analyses were correctly conducted with respect to genetically-defined ancestry groups. Accordingly, it would be more appropriate to refer to the study as "multi-ancestry". The term "ethnic" can have different meanings depending on the socio-cultural context.***

We thank the reviewer for their helpful feedback and appreciate that they find our work to be an “interesting study” and our analyses to be “well designed”. We agree about the importance of using accurate language when describing genetic ancestry and have changed both the title of the article and text within to refer to “multi-ancestry” analyses.

***2. It is not clear whether the weights to predict genetically-regulated C4 expression were derived from ancestry groups or whether they were derived from individuals of European descent. This should be clearly reported. If European-descent weights were used across the ancestry groups, this should be reported as a limitation.***

We apologize that our methods were not more clear. The multi-ancestry reference panel from Kamitaki et al., 2020 was used to impute *C4* structural alleles in the ABCD and UK Biobank datasets. *C4* GREx was then calculated using weights from Sekar et al., 2016, which is comprised of primarily European ancestry donors. As described in our original submission, we validated the accuracy of these weights in predicting *C4* expression in diverse ancestries by testing the relationship between predicted *C4A/C4B* expression and observed *C4A/C4B* expression in 340 African, 50 Latinx, and 863 European ancestry samples from the PsychENCODE dataset. As shown in Supplementary Figure 1A, observed *C4* gene expression is strongly associated with corresponding predicted gene expression in donors of African, Latinx, and European ancestries, except for *C4B* gene expression in African ancestry samples (P > 0.05). Thus, we have already validated the use of the Sekar et al. expression weights for imputing *C4* GREx multi-ancestry samples (please see Methods section entitled, “Validation of expression weights for C4 genes in PsychENCODE”).

We agree with the reviewer that these validation results (which appear in the Supplementary Information) were not adequately referenced in the main text and have updated the revised manuscript to include the following:

*“We validated the accuracy of C4 imputation weights for predicting gene expression in ancestrally diverse cohorts using data from 340 AFR, 863 EUR, and 50 Latinx donors from the PsychENCODE Consortium (Supplementary Fig. 1).”*

***3. It would be better to report the power analysis at the beginning of the results to show that the null results reported are not due to a lack of power.***

We agree and have moved the power analysis to the beginning of the results section under the heading “*Adequacy of statistical power to detect GREx-phenotype associations*”.

***4. It would be interesting to investigate whether there are differences in the C4 associations among individuals with high vs. low non-MHC SCZ PRS with respect to psychotic-like experiences and childhood cognition.***

This is a great suggestion. In the previous version of the manuscript we indeed assessed for the impact of the interaction of *C4A* and non-MHC SZ PRS, exactly as the reviewer requests (see Supplementary Table 11). We find that the interaction between *C4A* GREx and non-MHC SZ PRS is not a significant predictor of the number of psychosis-like events as indexed by PB-Q number, PB-Q severity, or binary psychosis status in European ancestry ABCD youth, or in females and males when assessed separately (p>0.05). We now make these analyses more explicit in the Results section of the revised manuscript:

*“Similar to previous findings (35), we did not observe a significant association between schizophrenia PRS and psychotic symptoms in youth – nor did we observe an interaction between C4A GREx and schizophrenia PRS in predicting PQ-B_sym_, PQ-B_sev_, or PP_bin_ in ABCD youth of EUR ancestry or when examining EUR females and males separately (Supplementary Table 11).”*

***5. Although the study is focused on genetically-regulated C4 expression, it would be interesting to include information regarding the association of C4 haplotypes with the phenotypes investigated. This would permit the assessment of the convergence between***

***transcriptomic and genetic associations*.**

The reviewer suggests a very interesting analysis investigating the impact of C4 haplotypes on childhood neurodevelopmental phenotypes. We agree that including such an analysis would aid in the assessment of convergence between transcriptomic and genetic associations with the C4 locus and in assessing the potential for age-related effects. As previous studies linking C4 haplotypes to phenotypic outcomes have focused on schizophrenia-related outcomes (e.g., Gracias et al., 2021; Mariaselvam et al., 2021; Sekar et al., 2016), we focused our assessment on psychosis-like experiences in the ABCD youth. Regression analyses were performed for the joint model assessing the impact of 14 unique allelic combinations on psychosis-like experiences (i.e., PQ-B_sym_, PQ-B_sev_, or PP_bin_) in the multi-ancestry ABCD sample. As previous research suggests that individuals with the BS allele have reduced *C4A* expression and lower risk for schizophrenia relative to other alleles (Sekar et al., 2016), the BS:BS haplotype factor level was set as the reference. In agreement with previous literature suggesting increased risk for schizophrenia in carriers of *C4A* long alleles (i.e., AL), youth with AL:AL haplotypes had higher number (PQ-B_sym_ 𝛽 = 0.50, p = 0.046) and severity (PQ-B_sev_ 𝛽 = 0.88, p = 0.02) of psychosis-like experiences at the baseline timepoint. The AL:AL haplotype was also a significant predictor of psychosis case/control status (PP_bin_ 𝛽 = 2.38, p = 0.042). Importantly, however, after adjusting for the number of tests performed (critical p-value = 0.05/3), these associations were no longer significant. Overall, these supplementary analyses indicate general concordance with the adult literature showing an effect of the *C4A* long allele on schizophrenia-associated symptoms. We have included the results of this supplementary analysis in the revised manuscript (Supplementary Fig. 12). We have added the following text to the revised manuscript:

*“Finally, to assess the convergence between C4 transcriptomic and genetic associations, regression analyses were performed for the joint model assessing the impact of 14 unique C4 allelic combinations on childhood PLEs. The C4 AL:AL haplotype showed nominally significant associations with greater PLEs; however, after Bonferroni correction results were no longer significant (Supplementary Fig. 15).”*

***6. The authors should discuss their findings with respect to previous studies that investigated C4 with respect to psychiatric disorders other than SCZ (e.g., PMID: 31570195).***

We thank the reviewer for this suggestion. A discussion of the relationship between C4 haplotypes and other disorders in now included in the revised manuscript:

*“In addition to the robust genetic association signal observed between schizophrenia and the major histocompatibility complex (MHC) locus, this region also shows associations with depression (Howard et al., 2018; Wray et al., 2018) and to a weaker extent bipolar disorder (International Schizophrenia Consortium, 2009; Ruderfer et al., 2013). However, in both depression and bipolar disorder, dissecting the MHC signal using fine mapping techniques suggests that the observed association is not driven by genetic variation in C4 haplotypes and may arise from other genetic variants in the region (Glanville et al., 2020; Mullins et al., 2021). Our null phenome-wide association study (PheWAS) testing the effects of the C4 locus on childhood psychiatric phenotypes agree these findings, suggesting that other sources of genetic variation apart from the C4 locus are likely influencing childhood cognition and behavior. Further large-scale studies will be needed to disentangle the genetic signal between MHC locus and diverse psychiatric disorders, and to examine the effects of other MHC-associated variants on neurodevelopmental trajectories.”*

**Reviewer 3:**

***In this study, Hernandez et al. use models of genetically regulated expression of C4 to study its relationship to youth neuropsychiatric phenotypes, as well as structural MRI derived brain indices. This study is rigorously performed and the finding of an association with the surface area of entorhinal cortex is interesting, however, I have a few suggestions that I feel would improve the manuscript.***

We thank the reviewer for their comments and assessment that our work was “rigorously performed” and that our results are “interesting”.

***Major Comments:***

***1. Page 10: The authors found evidence that youth in the bottom quartile of entorhinal SA were more likely to report psychotic-like experiences at 1-year follow up. I think the authors should also test and report the results of using entorhinal surface are as a continuous predictor, given that comparing the top and bottom quartiles will be a more exaggerated effect.***

We have taken the reviewer’s suggestion and performed regression analyses using entorhinal cortex SA as a continuous predictor in male and female youth. Generalized linear models with gamma family and log link were run using the glm package in R version 3.6.3. Fixed effects included age, socioeconomic status (SES; taken as the average of parent education and income at the baseline time point), four genetic principal components, and MRI device serial number (in accordance with the GREx-brain association reported in the previous version of the manuscript). We have added the following to the Results and have updated the code and associated GitHub repository to reflect these additional analyses.

*“Parallel regression analyses modeling entorhinal cortex SA as a continuous predictor were significant in females only, such that smaller entorhinal cortex at baseline predicted greater frequency and severity of PLEs at the 2-year follow-up (PQ-B_sym_ 𝛽 = -0.001, 95% CI [-0.002, -0.0001], P=0.03; PQ-B_sev_ 𝛽 = -0.002, 95% CI [-0.003, -0.0005], P=0.004).”*

***2. Do the authors have a sense how much the C4 variation is captured by GREx?***

We apologize, but it is unclear which specific aspect of C4 variation the reviewer is referring to. As shown in Supplementary Fig.1, we validate that *C4A/C4B* GREx is significantly associated with measured *C4A/C4B* expression in the PsychENCODE dataset. Imputed GREx accounts for 31.37% (adjusted r-squared) of the true C4A brain expression and 13.71% (adjusted r-squared) of the true C4B brain expression in European PsychENCODE donors between the ages of 5-15 (Supplementary Fig. 1B). All of these associations replicate the original work by Sekar et al., 2016, showing that C4 expression is associated with inherited C4 genetic variation.

***3. Page 10: At the 2-year follow-up there is no mention of the effect in males, was there a difference in psychotic-like experiences as seen in females at this time-point?***

There was not a significant association between entorhinal cortex SA and psychosis-like experiences in male youth at the 2-year follow-up. Effect size estimates and p-values for this association are presented in Supplementary Table 10. As the reviewer suggests, we have now plotted the distribution of PLEs by sex and performed a two-sample Kolmogorov-Smirnov test to evaluate whether the two samples (female and male) come from the same distribution. We find that for both measures, the Kolmogorov-Smirnov test is not significant, indicating that the data from female and male youth come from the same distribution (PQ-B_sym_ p=0.32; PQ-B_sev_ p=0.99). In addition, we tested whether the population mean ranks differed using a Wilcoxon signed-rank test; results were not significant, indicating that there were no significant differences between sexes (PQ-B_sym_ p=0.48; PQ-B_sev_ p=0.98).

We now more explicitly state the lack of associations in male youth under Results:

*“There were no significant associations between entorhinal cortex SA and PLEs in male youth at the 2-year follow-up.”*

***4. Given that the C4 GREx was not associated with psychotic-like experiences by itself, I think that the authors should be more mindful of the fact that there will be many other factors that will influence entorhinal SA, and thus, be careful as to not overstate the relationship between C4 and subsequent PLEs. As a result, I suggest that the authors should report the variance explained in entorhinal SA by the C4 GREx and compare that to other relevant phenotypes collected in this cohort. This would allow the reader to contextualise the strength of the effect of C4 related variation versus other factors.***

We thank the reviewer for bringing up this important point. We now report the variance explained in entorhinal cortex SA (change in model marginal r-squared) for *C4A* GREx compared to a host of other relevant phenotypes. As the reviewer hypothesized, we find a significant association between entorhinal cortex surface area and other relevant anthropometric and behavioral traits, including standing height, crystalized cognition, socioeconomic status, weight, total cognition, waist circumference, and weekend screen. *C4A* GREx explained more variance in entorhinal cortex surface area as compared to waist circumference and weekend screen time, but relatively less variance in comparison to the other variables tested. Results of these analyses are now provided in Supplementary Figure 16.

***5. Related to the above, is there is a reason that the authors only tested whether the entorhinal SA was associated with PLEs? One could probably also justify performing the same pheWAS of entorhinal SA, which naturally would have a higher multiple-testing burden.***

The reviewer brings up an interesting point. We agree that this question will be important to investigate in future studies. However, given the well-established clinical associations between C4 and schizophrenia, here we focused specifically on the phenotypic effects of C4A haplotypes, and on the effects of entorhinal cortex SA on childhood psychotic-like experiences in order to keep our analysis focused and to control for multiple comparisons, as acknowledge by the reviewer.

***6. It would be interesting to see if entorhinal surface area partly mediates the association of C4 GREx with the lack of planning subscale for which C4 demonstrated an association after multiple-testing correction in the pheWAS.***

We have run the mediation analysis as suggested by the reviewer. The effect of *C4A* GREx on the lack of planning subscale was partially mediated by entorhinal cortex size. The regression coefficient between *C4A* GREx and the lack of planning subscale and the regression coefficient between entorhinal cortex surface area and the lack of planning subscale was significant. The indirect effect was -0.014. We tested the significance of this effect using the *mediation* package in R. Proportion of the effect of *C4A* GREx on the lack of planning subscale that goes through entorhinal cortex surface area (i.e., the proportion mediated) was 0.029; the indirect effect was significant (P=0.006). We now report the following under Results:

*The only association that surpassed correction for multiple testing in the multi-ancestry cohort was between C4A GREx and the UPPS-P Impulsive Behavior Scale lack of premeditation/planning subscale (𝛽 = -0.05, 95% CI [-0.07, -0.04], P_FDR_ < 0.004), suggesting that youth with higher C4A GREx show less impulsivity and more careful thinking before taking action. This effect was partially mediated by entorhinal cortex size (indirect effect -0.014, P=0.006).*

***7. Perhaps the authors could consider testing whether there is an interaction with a cortical surface area polygenic score with C4, rather than just the schizophrenia PRS.***

As the reviewer suggests, we tested whether the interaction between surface area PRS and *C4A* GREx was a significant predictor of entorhinal cortex surface area at baseline. Surface area PRS were calculated in PRC-CS using the largest available GWAS from ENIGMA (Grasby et al., 2020). The interaction was not significant (𝛽 = -5.09, P = 0.11), indicating a dissociable effect of genetic variation at the C4 locus on entorhinal cortex structure that is independent of polygenic effects on brain surface area. These null findings are reported in the revised manuscript.

*We also tested whether the interaction between brain surface area polygenic scores and C4A GREx predicted entorhinal cortex SA; the interaction was not significant (P>0.05). Together, these findings indicate that the effects of C4A GREx on entorhinal cortex SA are specific to the C4 locus and are distinct from the effects of genetic liability for schizophrenia and adult brain size.*

***8. The discussion would benefit from more discussion about the neuropathology / neurobiology of the entorhinal cortex.***

We appreciate this suggestion. We have added the following information about entorhinal cortex neuroanatomy to the discussion.

*“The entorhinal cortex serves as a relay for corticohippocampal interactions, serving as the major input and output structure of the hippocampus (Munoz and Insausti, 2005). Recent high-field magnetic resonance imaging (MRI) research in humans suggests an anteroposterior division of entorhinal cortex functional connectivity, with anterior regions connected to anterior temporal brain areas critical for recognition memory, emotional processing, and social cognition, and posterior regions connected to posterior medial brain areas that support episodic memory and spatial navigation (Schroder et al., 2015; Ranganth and Ritchey 2012).”*

***9. There should be more discussion of the limitations of this study, I feel this is a bit lacking.***

A paragraph on limitations is not included in the revised manuscript.

*“This study has several important limitations. First, our analyses were restricted to youth within the early to mid-adolescent age range. It remains unknown the extent to which C4A GREx may affect neurodevelopment during infancy and very early childhood, as well as during adolescence, when many disorders of mental health – including schizophrenia – begin to emerge. Second, our PheWAS analyses were limited to a single neuroimaging modality, and it is unclear whether genetic risk for schizophrenia on the C4 locus may impact measures of brain functional and structural connectivity, as well as the developmental timing with which these associations may occur. Third, while there are many environmental variables that are likely to influence brain and behavioral development, the current study did not examine the relationship between C4A GREx and environmental factors known to increase risk for schizophrenia, such as smoking. It will be crucial for future studies to examine the impact of C4A expression on trajectories of brain development using multimodal neuroimaging techniques and incorporating potential interactions with environmental moderators.”*

***10. I also think that the authors overstate the utility of this finding in the concluding statement "Overall, these data provide a mechanistic, genetic basis for the identification of brain-based biomarkers predictive of psychosis risk"***

In line with the reviewer’s suggestion, we have reworded this sentence as follows:

*“Overall, these data help to identify genetically driven brain-based biomarkers predictive of psychosis risk.”*

***Minor Comments:***

***1. Line 14 - the authors refer to the MHC as the strongest schizophrenia association. The authors should specify that this is the most statistically significant association, there are larger effect size rare and structural variants (e.g. SETD1A, 22q11.2 deletion).***

As the reviewer suggests, we have changed to test to refer to the MHC as *“the most statistically significant genetic association with schizophrenia.”*

***2. The authors should explain more how the C4 GREx are calculated in this manuscript. The*** ***methods for this are hidden in the supplementary of the Sekar et al. paper and I think it's important to emphasise to the reader that this is not a GREx constructed of single SNPs like in conventional TWAS but is based on C4 structure.***

We have added more detailed information about how C4A GREx was calculated in the Methods section:

*C4 structural alleles were imputed for the ABCD dataset using Beagle4.1 (Browning et al., 2016) with the latest multi-ancestry C4 imputation reference panel composed of 1,265 samples of diverse ancestry (Kamitaki et al., 2020). C4A and C4B genetically regulated expression (GREx) was predicted using previously described expression weights (Sekar et al., 2016). C4 structural allele dosages were multiplied by the following coefficients to compute GREx:*

*C4A GREx = (0.47 * AL) + (0.47 * AS) + (0.20 * BL)*

*C4B* *GREx = (1.03 * BL) + (0.88 * BS)*

**Second round of review**

**Reviewer 1**

I am happy that my reviewer comments have been satisfactorily addressed in the revised manuscript.

**Reviewer 2**

The authors adequately addressed my previous concerns.
